# TES Nanoemulsions: A Review of Thermophysical Properties and Their Impact on System Design

**DOI:** 10.3390/nano11123415

**Published:** 2021-12-16

**Authors:** Eugenia Teodora Iacob-Tudose, Ioan Mamaliga, Alexandru Vasilica Iosub

**Affiliations:** Department of Chemical Engineering, “Cristofor Simionescu” Faculty of Chemical Engineering and Environmental Protection, “Gheorghe Asachi” Technical University of Iasi, 73 Prof. Dr. Docent D. Mangeron Blvd, 700050 Iasi, Romania; imamalig@ch.tuiasi.ro (I.M.); alexandru-vasilica.iosub@student.tuiasi.ro (A.V.I.)

**Keywords:** nanoemulsions, thermo-physical properties, nanodroplets concentration, temperature, dimensionless groups, heat transfer mechanisms, thermal storage operation

## Abstract

Thermal energy storage materials (TES) are considered promising for a large number of applications, including solar energy storage, waste heat recovery, and enhanced building thermal performance. Among these, nanoemulsions have received a huge amount of attention. Despite the many reviews published on nanoemulsions, an insufficient number concentrate on the particularities and requirements of the energy field. Therefore, we aim to provide a review of the measurement, theoretical computation and impact of the physical properties of nanoemulsions, with an integrated perspective on the design of thermal energy storage equipment. Properties such as density, which is integral to the calculation of the volume required for storage; viscosity, which is a decisive factor in pressure loss and for transport equipment power requirements; and thermal conductivity, which determines the heating/cooling rate of the system or the specific heat directly influencing the storage capacity, are thoroughly discussed. A comparative, critical approach to all these interconnected properties in pertinent characteristic groups, in close association with the practical use of TES systems, is included. This work aims to highlight unresolved issues from previous investigations as well as to provide a summary of the numerical simulation and/or application of advanced algorithms for the modeling, optimization, and streamlining of TES systems.

## 1. Introduction

With the increased energy demands of modern society, scientists have had to orient their efforts towards more efficient ways to store energy and to re-use it. New international requirements call for an improved exploitation of unconventional resources, including solar, geothermal, and wind energy, by confinement in thermal systems. The demand for thermal energy for both domestic and industrial applications has increased. Additionally, recent investigations seem to point to the fact that certain types of energy—for example, wind energy—collected in heat systems based on thermal storage materials enhance decarbonization by having an increased use efficiency [1]. Furthermore, thermal energy storage (TES) technologies are highly efficient and cost effective. In the next few years, the TES global market is expected to increase steadily; therefore, viable thermal storage systems are in increasing demand.

The storage principle relies on the ability of a system to absorb and store energy before releasing it whenever it is needed. Subsequently, thermal storage systems can be used to deposit energy for days, weeks, or even months in order to address night/day or seasonal irregularities related to supply and demand. Heat storage can be achieved by changing the internal energy of materials—namely, sensitive heat, latent heat, chemical heat, or a combination thereof. Thermal energy storage is based on the amount of heat absorbed or released by a material, with the efficiency of the process being characterized by the storage capacity. The highest values have been recorded for chemical storage (200 KWh/t), followed by phase transformation (50–150 KWh/t) and sensitive heat (10–50 KWh/t), all having specific advantages and disadvantages [2]. 

Due to the current trend for exploiting available energy more judiciously, TES nanomaterials, with much improved thermo-physical properties, offer a viable solution for increasing energy storage and using it in many domestic as well as industrial sectors [3,4]. At the same time, these systems offer new research opportunities—on the one hand, due to the much-needed technologies for further improving their physical properties, and on the other hand, due to the necessity of designing new materials with features that are increasingly adapted to specific requirements. Their integration within a functional system, followed by their optimization and rigorous control, is another outstanding step in the development of TES. 

Among the materials applied for the storage and reuse of thermal energy, nanoemulsions/nanosuspensions have a significant place. In the past thirty years, these fluids have been extensively investigated, especially with regard to their application in the pharmaceutical, cosmetics, and food industries, which have characteristic requirements, such as tunable stability, specific rheologic properties, high bioactivity, or added nutritional value. However, the criteria for their thermal storage applications are different and the working conditions are also distinct. The current energy needs have increased researchers’ interest in using nanosuspensions/nanoemulsions as flexible thermal storage systems, especially in recent years. Thus, several studies have concentrated on finding and characterizing new suitable two-phase stable nanosystems or improving their properties for TES applications. Nevertheless, there are a number of issues that need to be highlighted and further investigated. Furthermore, the simple consideration of their properties at the nanoscale level, without providing an extrapolation at the macroscale level, is inadequate. Therefore, this review has several objectives related to nanoemulsions used as thermal storage systems:

- The detailed description of the thermo-physical properties of nanoemulsions and their dependence on significant parameters, including a discussion of standard techniques, less customary measurement methods, and available literature equations based on theoretical thermodynamics, classical mechanics, or other theoretical foundations. We also aim to make a comparison to available experimental data and discuss critical points of view related to these issues.
- The analysis of major dimensionless numbers and proposed heat transfer correlations in close connection with occurring heat transfer mechanisms depending on the type of nanoemulsion used and the enclosure geometry, which influences the system operation and performance.
- Establishing possible gaps or missing links in property measurement and interpretation at the nanoscale level that equally, at the macroscale level, have a major impact on system design and control.

### 1.1. TES Requirements and Materials 

Thermal energy storage materials designed for sensible heat storage, especially at high temperatures (usually above 150 °C), have to fulfill a number of requirements related to their physical properties; for example, they must have a high density, low vapor pressure, high values of specific heat and thermal conductivity, high chemical stability and compatibility with the container materials, low toxicity, high availability, and lack of fire or explosion hazards. They must also be non-polluting and cost-effective [5]. 

Sensible heat storage (SHS) equipment stores energy by raising the material temperature according to the equation:

(1) $$E_{SHS}=mc_{p,SHS}\left|\left(T_{i,SHS}-T_{f,SHS}\right)\right|$$

where *m*, $c_{p,SHS}$, $T_{i,SHS},$ and $T_{f,SHS}$ are, respectively, the mass, specific heat, initial temperature, and final temperature of the SHS material. For this type of system, the more the temperature is increased, the greater the heat loss will be, meaning that more insulation is needed, which raises the SHS costs. Thus, to further increase the amount of energy stored, a larger mass of storage material must be used, leading to an increase in the equipment size. In conclusion, the SHS equipment is less effective at storing large amounts of energy within a limited space [6]. 

**Figure 1:**
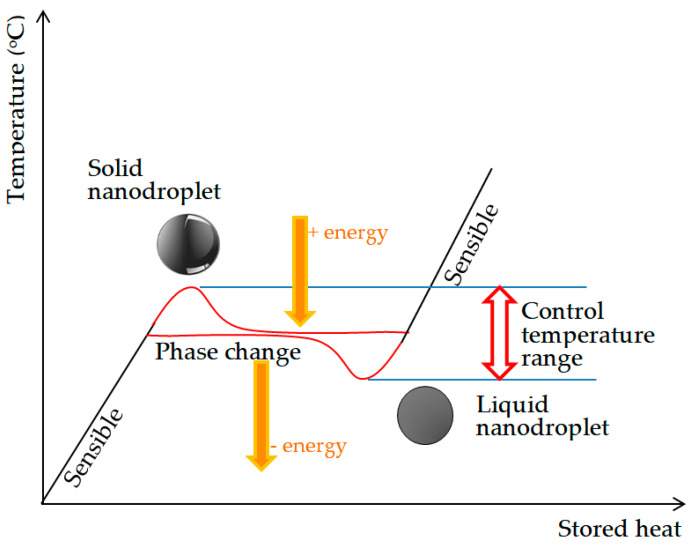
Sensible and latent heat cycle when using storage SHS and LHS materials.

The phase change materials used for thermal energy storage are usually based on solid–liquid phase change but can also be based on solid–solid or liquid–gas phase changes depending on the material used and the working temperature interval of the storage equipment. The technology implies a charging process, whereby the material melts and heat is stored at a constant temperature, followed by a discharging phase, where the material solidifies at a constant temperature and heat is released, as presented in Figure 1. The cycle is repeated as many times as needed [6,7,8,9]. 

In this case, PCMs have to possess physical characteristics such as a low density variation, small volume change, no phase separation, high energy density, reduced or no supercooling, high chemical stability, large phase change enthalpy and specific heat, high thermal conductivity, high thermal stability, and reproducible phase change after repeated cycles [6,10]. The characteristics of availability, non-toxicity, low environmental footprint, and cost effectiveness complete these requirements. PCM-in-water emulsions, hydrate slurries, and microencapsulated stabilized PCM suspensions are the three primary forms of phase change slurries used for thermal control [11]. 

A latent heat storage (LHS) system using a phase change material accumulates and releases energy according to the equation:

(2) $$E_{LHS}=m_{LHS}\left[c_{pi,LHS}\left|\left(T_{i,LHS}-T_{mt}\right)\right|+∆H_{f}+c_{pf,LHS}\left|\left(T_{mt}-T_{f,LHS}\right)\right|\right]$$

where $m_{LHS}$, $c_{pi,LHS}$, $c_{pf,LHS}$*,* $∆H_{f}$*,* $T_{i,LHS}$*,* $T_{f,LHS}$*,* and $T_{mt}$ are, respectively, the phase change material mass, the initial phase and final phase-specific heat capacities, the latent heat energy, the initial temperature, the final temperature, and the melting temperature. 

Latent heat storage systems can undergo solid–solid, solid–liquid, or liquid–gas (vapor) phase transformations depending on the type of the material used [8,12]. 

The highest energy storage density pertains to liquid–gas systems; nevertheless, these systems usually undergo large volume changes, resulting in larger storage volumes and sometimes in pressure building, which implies the usage of robust tanks and thus increased costs. 

Solid–liquid materials undergo much smaller volume changes, usually ~10% or even less, despite their lower phase transition heat when compared to solid–gas systems. They are characterized by small temperature differences between the charging and discharging cycles and a relatively high total heat capacity that translates into a reduced volume and weight of the storage units. Thus, they are becoming extremely attractive, both practically and economically. In this case, the phrase ‘phase change materials (PCMs)’ refers to low molecular compounds of an inorganic (salts, salt hydrates, hydroxides, or alloys) or organic nature (paraffins, alcohols, fatty acids, esters [13,14,15,16,17,18,19], etc.), polymers (PEG) [20,21,22,23], and nanoemulsions (paraffins-in-water, beeswax-in-water, water-in-polyalphaolefine, etc.). Among the most broadly used phase change materials are paraffins—i.e., saturated hydrocarbons with C5-C15 in a liquid state and >C15 solids—which have been intensively studied [24,25,26,27,28,29,30] and used as PCMs due to their high latent heat storage capacity. Despite their lower thermal conductivity, which reduces the rate of storage and release cycles [31], they have a variable molar mass and diverse phase change temperatures, making them suitable for different applications. They present a high structure stability and thus constant thermal properties even when passing through a large number (1000 or more) of charging/discharging cycles [26,30]. Furthermore, paraffins do not induce corrosion in metallic containers; they are non-toxic and environmentally harmless [12]. 

Solid–solid latent heat systems use the heat associated with the phase transition of one crystalline form to another and have a lower heat phase transition than solid–liquid PCMs; their major advantage is the lack of leakage at higher temperatures than the phase transition temperature, in comparison to solid–liquid systems [32,33]. These PCMs comprise low molecular compounds such as polyalcohols or polymers—for example, crosslinked polyethylene, polyurethanes, polybutadiene, and modified poly(ethylene glycol) [34,35,36,37]. 

### 1.2. Nanoemulsions as TES Fluids: Advantages

Nanoemulsion fluids are heterogeneous systems belonging to a broad class of multiphase colloidal dispersions. They usually contain two immiscible phases—one dispersed in another—stabilized by amphiphilic surfactant molecules. For thermal storage systems, the use of nanoemulsions offers numerous advantages. The dispersion of liquid droplets stabilized by a surfactant (in order to reduce coalescence, aggregation, and further particle interactions) within another heat transfer liquid further enhances the heat transport due to their high mobility and diffusivity [38,39]. The small volume changes and temperature differences between their charge/discharge cycles; high surface area per unit volume, sometimes exceeding 10 m^2^/cm^3^ [40]; and enhanced thermo-physical properties, such as thermal conductivity, specific heat capacity, or latent heat (leading to the increased heat transfer efficiency of the storage system, increased availability, and reduced costs), define nanoemulsions as materials with great potential in TES and heat enhancement applications [11,12,13,22,23,24,25,26,27,28,29,30]. 

The nanofluids class, which contains other nanomaterials intended for thermal storage, comprises solid nanoparticles suspended in a continuous liquid phase, and these have also been intensely studied [41,42,43,44,45,46]. Their superior thermal properties can be explained based on different mechanisms, especially Brownian motion and their increase with increased temperature and nanoparticle concentration, which prove that they are also suitable candidates for heat transfer applications. However, nanofluids’ particle concentrations can only be increased within a narrow range, mainly due to the reported sedimentation inconvenience. Conversely, in case of nanoemulsions, nanodroplets’ concentrations and sizes can be varied within larger ranges due to the closer density values of the constituent phases, resulting in enhanced convection and heat transmission capabilities. 

In the last 2–3 years, several nanoemulsions fulfilling the above-mentioned TES material requirements have been validated for use in different heat storage applications [47,48,49,50,51,52]. Additionally, several composite or hybrid materials have been studied and proposed as PCMs with improved thermo-physical properties [53,54,55,56].

Despite the fact that nanoemulsion fluids are also prone to undesired effects, such as particle clustering, bridging, or other mechanical interactions, which obviously need to be further investigated and require novel solutions, the large number of studies and findings [12,30,57,58] in this area underlines their versatility in thermal handling and storage applications.

## 2. Nanoemulsions Overview 

### 2.1. General Aspects

As already stated, nanoemulsions/nanosuspensions are two-phase dispersions formed of nanosized gas, liquid, or solid particles distributed throughout a continuous liquid phase. 

Throughout this study, distinctions should be made between the terms nanoemulsions and nanofluids, the latter being used to denominate nanosuspensions with application in thermal storage. 

The average reported emulsion droplet diameter is <200 nm [59,60]; other studies have reported values within the range 20–500 nm, with a low polydispersity [50]. There is no cutoff value for the diameter of nanodroplets that can differentiate a nanoemulsion from an emulsion, since physical changes occur gradually from one system to another. Nanoemulsions are kinetically stable but thermodynamically unstable [61] due to the molecular interactions occurring between the oil–water interface and the driving force that tends to reduce the contact area between the two phases. As a consequence, nanoemulsions can be unstable over large time periods, and phenomena such as sedimentation, creaming, flocculation [59,62], coalescence (Ostwald ripening) [63,64], or phase separation may occur. To prevent these unwanted effects, besides emulsifiers, texture modifiers can be added for the alteration of rheological properties, weighting agents can be added to match the density of the dispersed phase to the continuous phase density [62] and inhibit gravitational separation, and ripening inhibitors can be added to retard droplet growth. The role of emulsifiers is highly significant and numerous studies have addressed it [65]. Besides reducing the interfacial tension, when emulsifiers are adsorbed onto the oil–water interface to facilitate droplet rupture, they prevent subsequent aggregation. The capacity of emulsifiers to stabilize nanoemulsions varies greatly; therefore, it is critical to determine which one is best for a certain application. For PCM nanoemulsions, the surfactant–phase interaction becomes important within a specific range of temperatures. Several changes occurring in the progress of thermal properties during charge/discharge cycles have been attributed to the behavior of emulsifiers within certain working conditions; these are discussed further in Section 3. 

At the laboratory scale, nanoemulsions can be obtained based on low- or high-energy processes [66]. Usually, low-energy methods require certain physicochemical properties of the components and specific compositions, thereby limiting their application. However, recent studies have reported highly stabilized phase change nanoemulsions despite the use of low-energy preparation techniques [67]. In high-energy methods, large disruptive stresses are supplied by technical devices such as ultrasonicators, microfluidizers, high-shear stirrers, high-pressure homogenizers, rotor/stator systems, etc., in order to force the nanodispersion of one phase into another with the formation of huge interfacial areas [68]. Therefore, high-pressure homogenizers are the most widely used devices for preparing nanoemulsions [68]. Moreover, producing nanoemulsions using ultrasounds is a cost-effective process that requires less use of surfactants [69,70] and creates, depending on the sonication characteristic parameters, more uniformly dispersed and thus more stable nanoemulsions [71]. The scientific literature offers some excellent reviews on preparation methods, including the main advantages/disadvantages associated with certain methods [61,62,72]. Some studies have reported results on commercially available thermal storage materials [19]. For practical applications, low-energy methods offer an attractive alternative. 

The impact of the preparation method used on the physical properties of nanodroplets is undeniable, as it directly affects their diameter, dispersion, polydispersity index, and subsequent mechanical interactions. Some major findings in this area are presented in the next section. 

### 2.2. Nanoemulsion Structure and Droplet Size 

Many parameters influence the microstructure of nanoemulsion fluids, including the surfactant type, dispersed liquid type, and their concentrations; the molar ratio of dispersed liquid to surfactant; and the temperature [73]. Controlling the final droplet size is a crucial part of nanoemulsion preparation that affects all of the features stated. Usually, nanoemulsions contain droplets of different sizes, so they are characterized in terms of the particle size distribution or the mean droplet diameter and polydispersity index. In consumer goods industries, nanoemulsions with small droplet sizes are highly desirable due to their low interfacial tension and, thus, higher bioactive release rate and absorption. Thermal storage nanoemulsions also require a high surface area, which is necessary for efficient heat storage and release. Depending on the preparation method used, a number of parameters, such as surfactant concentration, surfactant molecular geometry, temperature, mixing rate, relative viscosity of the dispersed phase with respect to the continuous phase, and addition rate, can affect the droplet size and dispersion:

- The surfactant concentration increase usually renders a minimum value for the mean droplet diameter and a low polydispersity index, which is a measure of the emulsion’s droplet size uniformity and stability. This trend has been reported for a large number of systems and NE preparation methods [74,75,76,77,78,79].
- The molecular geometry of a surfactant influences the curvature of the monolayer that the surfactant forms and influences the packing at the interface between the two phases, thus favoring the formation of either O/W emulsions or W/O emulsions, or even of bicontinuous systems [75]. Some surfactants that have been extensively used in different experimental studies, such as Tween 20, 40, 60, 80, and 85, and that are known to have related hydrophilic–lipophilic balance (HLB) numbers do not lead to NE particles with the same size due to their differences in molecular geometry. Usually, double bonds in non-polar chains of non-ionic surfactants favor NE particles with a smaller diameter.
- The temperature increase within different limits, depending on the system, may enhance the surfactant solubility in the oil phase, influencing the surfactant film curvature and promoting the formation of O/W nanoemulsions [59,80]. However, higher temperatures can cause the dehydration of the polar head group of nonionic surfactant molecules and a decrease in the solubility of the hydrophilic surfactant, inducing the leakage of the surfactant from the oil–water emulsion and, thus, the aggregation of the NE droplets [81].
- Some studies have underlined the impact of the mixing rate used on the droplet size, indicating that gentle mixing is needed to obtain very fine droplets [82,83]. Usually, the use of a low range mixing rate (~300 rpm) allows for the formation of fine droplets; a slight increase in the rate up to 600 rpm can reduce the droplet size due to the stronger energy conveyed, breaking the particles and providing a more uniform distribution of the surfactant–oil phase in the aqueous phase [80,84,85,86]. A significant increase in the mixing rate promotes destabilization, along with unwanted phenomena such as coalescence and sedimentation [84]. The width of the particle size distribution does not depend strongly on the mixing rate [82].
- The relative viscosity of the dispersed phase with respect to the continuous phase can also influence the particle size diameter. An increase in the dispersed phase viscosity and a decrease in the continuous phase viscosity leads to larger NE droplet sizes, as reported in [87].
- The addition rate should be slow enough to allow NE stabilization; however, if the addition time is very large, the nanoemulsion instability and droplet size will increase. On the other hand, an increase in the addition rate will lead to a higher mean droplet size [80].

Depending on the nanoemulsion preparation method used, other parameters can also influence the nanoemulsion structural characteristics [88]. However, for TES nanoemulsions, there are several factors affecting their subsequent physical behavior and properties, such as temperature and repeated charge/discharge cycles, which are further discussed in Section 3. 

## 3. Nanoemulsions’ Thermo-Physical Properties

As already mentioned, TES materials must be able to withstand certain conditions and must have certain physical characteristics. In the following, relevant thermophysical properties related to the nanoemulsions’ structure, the influence of parameters such as temperature and nanoparticle concentration, and their impact on thermal performance in different geometric configurations are discussed. Experimental techniques and theoretical equations based on valid molecular or classical mechanics theories, intended, respectively, for property measurement or computation, are of great interest for modeling by numerical simulation or using artificial intelligence techniques that can facilitate an efficient, low-cost approach to confirm the performance of nanoemulsions such as TES fluids in various configurations. 

Particle size, polydispersity index, zeta potential, stability, thermodynamic stability, viscosity, thermal conductivity, specific heat capacity, melting–solidification enthalpies, melting temperature, the temperature range of the phase change, and surface tension are some of the relevant physical properties for characterizing a nanoemulsion, both during and after repeated charging/discharging cycles, in order to assess its potential use. Characterization techniques range from very well-known standard methods, such as differential scanning calorimetry, thermal gravimetry, transmission electron microscopy, and small-angle neutron scattering [89,90,91], to laboratory techniques—for example, the T-history method [92] or the use of a survismeter. All of these can be considered equally important, since the first ones offer precise readings, sometimes of several thermal properties, and reduced errors (though at higher costs), while the others can be extremely useful for the fast simultaneous estimation of similar properties of several prepared nanoemulsions in a simple laboratory setup. 

The property measurement can also help to emphasize some other characteristics of a nanoemulsion. Sudden changes in a monitored property, such as the nanoemulsion’s electrical conductivity, can indicate the phase inversion temperature, which is defined as the temperature at which the dispersed phase becomes the continuous phase and vice versa [93,94,95,96]. 

### 3.1. Density

Density or mass per unit volume can easily be measured experimentally with picnometers [97], hydrometers [98], or oscillating U-tube density meters [99,100]. Usually, the sample density is measured right after preparation, since the physical state of an emulsion can change and, thus, errors may be induced [62]. The methods used for density measurement are non-expansive and versatile. 

**Figure 2:**
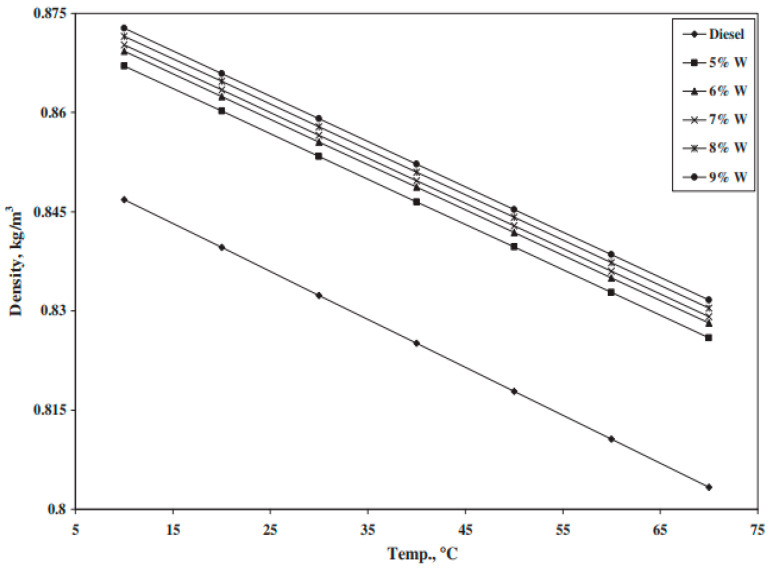
Density variation with dispersed phase concentration and temperature for a water-in-diesel nanoemulsion [101] (reproduced with permission from Elsevier).

Usually, studies report density measurements without carrying out an investigation of the dispersed phase concentration and/or influence of temperature on the properties. In the case of thermal storage nanoemulsions, such measurements, performed within the working temperature range, are essential, as the material will undergo many charging/discharging cycles. An example of the influence of the mentioned parameters on the density of a water-in-diesel nanoemulsion, with an expected density increase when the water concentration increases and density decrease when the temperature rises [101], is shown in Figure 2.

The nanoemulsion density can be written as:

(3) $$\rho_{NE}=\rho_{c}\left(1-\varphi \right)+\rho \varphi$$

where $\rho_{c}$ is the density of the continuous phase where the particles are dispersed, ρ is the droplet density, and φ is the volume fraction of droplets. This equation can be used to determine the volume fraction based on the experimental values for the densities involved, since these are easily measurable quantities. 

Density can clearly have an impact on the TES storage volume, as well as affecting the fluid convection to some degree, as explained later. 

### 3.2. Viscosity

Viscosity is related to the internal resistance of the fluid when flowing, simply due to the friction forces existing between the constitutive elementary volumes. The nanoemulsion viscosity depends on the temperature, particle size, and concentration, which are influenced by the preparation method used. 

The viscosity of nanoemulsions is a decisive factor in the pressure loss occurring during NE handling/conveyance and the power required for transport to the heat transfer equipment. 

#### 3.2.1. Experimental Measurement

Instruments such as cones and plates, coaxial cylinders, and parallel disks are mostly used to assess the NE rheological behavior and measure viscosity (Brookfield viscometer, Malvern Kinexus Pro rheometer, Hoeppler falling ball viscometer, Ferranti-Shirley viscometer, etc.) [30,58,102,103,104,105]. 

The viscosity of nanoemulsions can also be measured with a survismeter, which can be used to determine other NE properties, such as the surface tension, contact angle, dipole moment, and particle size of nanoemulsions [106]. The apparatus is reported to be able to conduct accurate measurements [107]; however, more data are needed to validate its use. 

The viscosity value is an indicator of the emulsion type; a low viscosity indicates a W/O type and a high viscosity indicates an O/W type [108]. Additionally, viscosity can be related to the emulsion stability, since it depends on its microstructure and particle mechanical interactions [73,109,110,111,112]; at the same time, any changes in the nanoemulsion structure can be highlighted by the changed viscosity values. 

#### 3.2.2. Correlations and Theoretical Analysis

Viscosity is an important parameter used for NE characterization that has been thoroughly investigated, both experimentally and theoretically, in both newtonian and non-newtonian nano-colloidal fluids. 

Some equations that are used for estimating the viscosity of nanoemulsions were created for viscosity calculations for nanosuspensions and can only be used if one assumes liquid droplets’ behavior to be the same as that of rigid solid particles (the viscosity ratio is defined as droplets’ viscosity/continuous fluid viscosity, $\lambda =\mu_{d}/\mu_{c}$—i.e., λ→∞). Some historically important proposed equations are included in Table 1; their premises, assumptions, and some of their limitations/drawbacks are discussed by Pal [113].

Many of the above equations are simplified forms of a power series equation, as presented below:

(15) $$\mu_{r}=1+C_{1}\varphi +C_{2}\varphi^{2}\pm C_{3}\varphi^{3}\pm \cdots$$

where $C_{1}$ can range from 2.5 to 5.5 depending on the particle shape (2.5 for spherical [114], larger or lesser than 2.5 for elongated or soft liquid particles [126], $C_{2}$ from 7.3 to 14.1, $C_{3}$ from 16 to 50 [127], with a second order expansion being valid for φ≤0.2 and a third order expansion being valid for 0.2≤φ≤0.4). These terms account for particle interactions, clustering, and other mechanical interactions. It was suggested that taking into consideration higher-order expansion terms can introduce more than 10% error in the viscosity ratio for a particle volume fraction of φ≤0.15 and, instead, that an exponential term in the form of *A exp* (*B* φ) should be used since the errors are diminished, with A and B coefficients determined based on experiments. 

Ford [117] also accounted for particle rotation inhibition and interlocking via an equation similar to Equation (15), through the fifth- and seventh-order terms, respectively:

(16) $$\mu_{r}=1-2.5\varphi +C_{2}\varphi^{5}-C_{3}\varphi^{7}$$

where $C_{2}=11.0$ and $C_{3}=11.5$. 

More recent studies on the viscosity of nanoemulsions have indicated that Equation (15), where $C_{2}=2.5,\;C_{3}=6.25$, and A and B are determined from experimental data representations, give comparable results [95,128] for a volume fraction of φ≤0.28. However, at φ≥0.41 there is a significant increase in the experimental data in comparison to that of the model; most likely, this is due to polydispersity and increased particle interactions and deformations. 

Most studies suggest that at nanodroplet concentrations above a certain value, they start interacting hydrodynamically or by means of colloidal forces [129]. If the shear stress is low, the particle distribution will be determined by the Brownian motion and, thus, the system will have a higher viscosity [130]. However, if the shear stress increases, the hydrodynamic forces will overcome the Brownian motion and the nanodroplets will be ordered along the fluid streamlines, meaning that the viscosity will decrease [131]. The electrostatic charge of the droplets determines repulsive interactions and has a greater impact on the NE viscosity, especially for small droplets which have a shell thickness comparable to their radius. When conditions for aggregation or flocculation occur, the NE viscosity will increase due to the entrapment of the continuous phase between the aggregated droplets. This effect was underlined by Mao and McClements [132], who found that the viscosity of nanoemulsions with a mixture of cationic and anionic droplets was higher than that of NE containing only cationic or anionic droplets. 

Later, based on the differential effective medium approach and particle crowding effects, Pal [133] derived two equations valid for *φ* < *φ_m_* for concentrated emulsions that can be reduced to either Mooney or Krieger and Dougherty equations (see Table 1, Equations (12) and (13)):

(17) $$\mu_{r}{\left(\frac{2\mu_{r}+5\lambda }{2+5\lambda }\right)}^{\frac{3}{2}}=\exp\left(\frac{2.5\varphi }{1-\frac{\varphi }{\varphi_{m}}}\right)$$

(18) $$\mu_{r}{\left(\frac{2\mu_{r}+5\lambda }{2+5\lambda }\right)}^{\frac{3}{2}}={\left(1-\frac{\varphi }{\varphi_{m}}\right)}^{-2.5\varphi_{m}}$$

Both equations are non-linear in their relative viscosity and require numerical solutions for property calculation. 

In some specific cases, certain viscosity models do not render results similar to the experimental data due to a significant effect that occurs besides particle interactions—namely, solvation. This effect consists of a nanoscopic solvation cell induction of approximately 2 nm at the colloidal particles/nanoparticles surface [134]. Using high-energy X-ray scattering, its presence was highlighted in the form of an additional signal—i.e., a damped oscillation for dispersed nanoparticles in comparison to dried nanoparticles, which can prove to be independent of the particle size or shape. This has a substantial effect on the nanomaterial’s rheology.

Accounting for the solvation effect, Pal [135] proposed an equation for the calculation of the nanosuspension’s relative viscosity:

(19) $$\mu_{r}={\left\{1-\left[1+\left(\frac{1-\varphi_{m}}{\varphi_{m}^{2}}\right)\varphi_{solv}\right]\varphi_{solv}\right\}}^{-2.5}$$

This is valid for *φ* < *φ_m_*, where *φ_solv_* is the volume fraction of solvated nanoparticles. If no solvation occurs—i.e., $\varphi_{s}\to \varphi$—the above expression will be reduced to the Roscoe equation (see Table 1, Equation (11)). 

The solvation effect and details related to the particle interactions and aggregation tendency within the continuous fluid have been discussed by Pal [136]. Different experimental data sets from the literature, several for nanoemulsions and several for nanosuspensions, consist of the relative viscosity measured for nanodroplets of different diameters, ranging between 27.5 nm and 205 nm, as a function of the volume fraction of solvated nanoparticles. These values are following the same curve, which is given by the next equation:

(20) $$\mu_{r}=\frac{1+\frac{3}{2}\varphi_{eff}\left(\frac{2+5\lambda }{5+5\lambda }\right)}{1-\varphi_{eff}\left(\frac{2+5\lambda }{5+5\lambda }\right)}$$

where $\varphi_{eff}$ is the effective volume fraction of the dispersed phase, which can be calculated based on:

(21) $$\varphi_{eff}=K\varphi_{s}=KK_{sol}k_{s}\varphi$$

where $\varphi_{s}$ has the same meaning as previously discussed, φ is the actual volume fraction of the unsolvated particles, *K* is the aggregation coefficient, and $K_{sol}$ is the solvation coefficient. The aggregation coefficient *k*, defined as the $\frac{\varphi_{eff}}{\varphi_{s}}=1/\varphi_{m}$ ratio, was investigated based on a number of proposed equations established after the graphical representation of the effective volume fraction–solvated particle volume fraction—i.e., $\varphi_{eff}$–$\varphi_{solv}$—was plotted. For nanoemulsions, the relative viscosity increases significantly with the increase in the viscosity ratio (0.01 ≤ *λ* ≤ 1000). The experimental results [30,102] confirmed the theoretical model for an assumed maximum packing volume fraction $\varphi_{m}=0.637$ for droplets with a spherical shape. Despite the good correlation between some of the experimental data in the literature and the proposed model (Equation (20)), further comparisons between the measured viscosities and this model would validate its general use. 

Based on theoretical thermodynamics, classical mechanics flow, nanoparticle–matrix interaction, and other effects, Machrafi [137] proposed a model that correlates the density and viscosity of nanofluids/nanoemulsions; this is given in a simplified form by Equations (22) and (23) for spherical and cylindrical particles, respectively:

(22) $$\mu_{n}=\mu_{c}\left(1+\frac{5}{2}\left(\frac{\varphi \rho_{p}}{\rho }+2\left({\left(1+\frac{\delta }{a_{p,sph}}\right)}^{2}-1\right)\frac{\varphi \rho_{p}}{\rho }\left(1-\frac{\varphi \rho_{p}}{\rho }\right)\right)\right)$$

(23) $$\mu_{n}=\mu_{c}\left(1+\frac{5}{2}\left(\frac{\varphi \rho_{p}}{\rho }+2\frac{\delta }{a_{p,cyl}}\frac{\varphi \rho_{p}}{\rho }\left(1-\frac{\varphi \rho_{p}}{\rho }\right)\right)\right)$$

where $\mu_{n}$ and μ are the nanofluid and the continuous phase viscosities, respectively; $\rho_{p}$ and ρ are the particle and fluid densities, respectively; δ is the nanolayer thickness around the particle; and $a_{p,s}$ and $a_{p,c}$ are the particle radius for spherical and cylindrical shapes, respectively. The model analytically establishes some relevant dependencies—for example, a higher nanoparticle density induces a higher particle–fluid velocity difference, meaning there is more drag force and, thus, an increased effective viscosity in the nano-system. Several experimental data for different nanofluids do compare well to the model. Additionally, an extrapolation for well-stabilized nanoemulsions, such as silicon oil–water, mineral oil–water, and water–mineral oil, gave good results and validated the model’s universality. 

Additionally, this model emphasizes the usage of density as a sufficient property for estimating the effective viscosity of nanofluids/nanoemulsions as a function of their content in nanoparticle or nanodroplet emulsions. 

#### 3.2.3. Influence of Dispersed Phase Concentration on Viscosity

**Figure 3:**
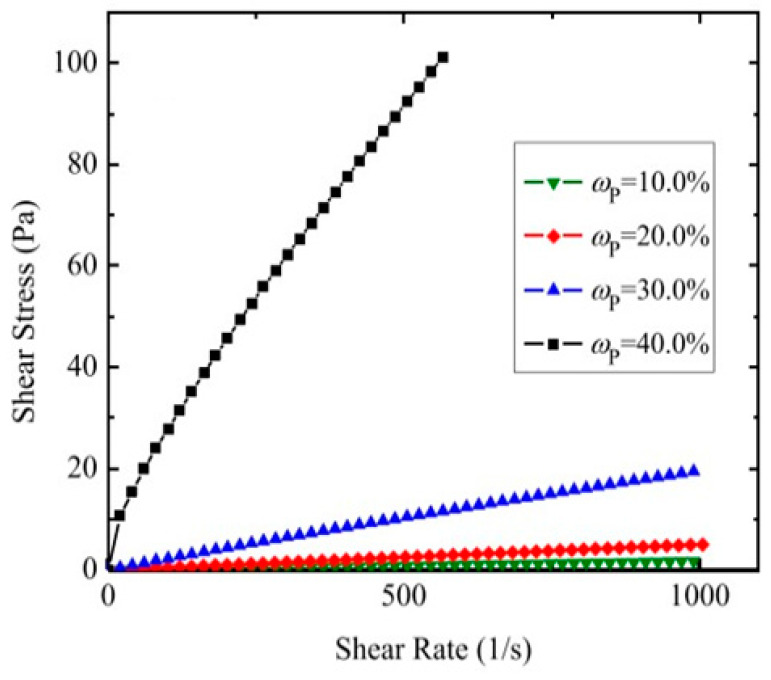
Variation in the shear stress with the shear rate of the n-hexadecane PCE at different mass fractions [58] (reproduced with permission from Elsevier).

The viscosity of nanoemulsions was found to increase with the increase in the dispersed phase concentration. For example, a 30% n hexadecane–water nanoemulsion, as presented in Figure 3 [58], was reported to have a 20-times-higher viscosity than water, and this was found to increase nonlinearly with the particle concentration. However, for emulsions with low concentrations ranging between 10 and 30%, this relationship was linear, which was most likely due to reduced interactions between nanodroplets. For a 33 wt% paraffin wax–water emulsion, Sivapalan [138] reported a 7- to 9-times-higher viscosity than that of water in the temperature range of 25–60 °C. The trends reported in different studies are similar, but their increases may be quite different, which can be attributed to possible differences in the nanoemulsions’ structure. 

Many PCEs are newtonian fluids that start exhibiting non-newtonian behavior for larger concentrations of the dispersed phase (usually >30%) when the shear stress–shear rate dependence becomes nonlinear [58,139], finding sustained by several studies, as shown in Table 2. When the shear stress is increased over a high limit, some PCEs experience a decrease in their viscosity, possibly due to the breakage of formed aggregates at lower shear stresses and droplet reorientations [131]. However, Cabaleiro considers the flow field deformation to be negligible in the case of nanoparticles [30] and instead credits the decrease in the viscosity to inner droplet convection and elastic collisions. 

Additionally, higher viscosities are registered for solid dispersed droplets than for the same liquid droplets [140,141]. 

Very few nanoemulsions have been reported to have atypical behavior due to increases in the particle concentration [57], exhibiting a maximum at a specific water particle concentration in water/polyalphaolefine (PAO) nanoemulsions, attributed to the nonlinear inner structure changes, revealed by small-angle neutron-scattering curves. 

The increase in viscosity with a more dispersed phase concentration can lead to a reduced fluidity [146], as well as to a decreased heat transfer coefficient due to the reduced level of convection happening within the system. 

#### 3.2.4. Temperature Influence on Viscosity

**Figure 4:**
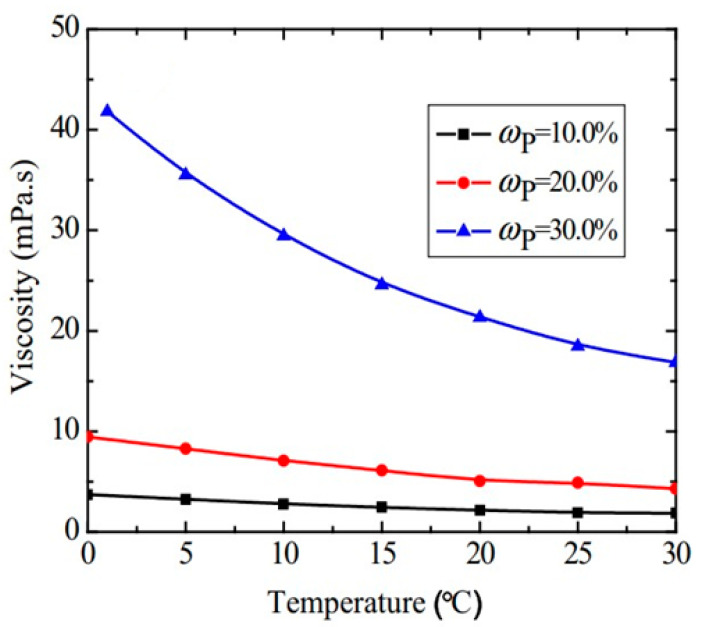
Variation in the viscosity of n-hexadecane PCE with temperature, in the shear rate range of 0 to 1000 s^−1^ [58] (reproduced with permission from Elsevier).

In nanoemulsions, the variation in viscosity with temperature has been investigated by a number of authors [57,58,138]. The decrease in viscosity with the increase in temperature was found to become more accentuated the higher the concentration of nanodroplets was, probably due to the extra energy provided increasing the Brownian motion and thus resulting in a reduction in viscosity [131,138,139,147]. Relevant results, showing the physics behind the decrease, are shown in Figure 4 [58]. This trend is favorable for heat transfer PCM applications since it leads to a lower pumping power in a fluid with enhanced thermal properties. 

For nanofluids, several studies have reported an exponential decrease in the viscosity with an increase in temperature [45,148], especially at increased particle concentrations (~6%). The trend for nanoemulsion fluids seems to be the same as that for nanofluids, even though less accentuated. This can have a larger impact on pumping power in the case of nanoemulsions compared to nanofluids; however, if the increase in the specific latent heat is significant, it may be economically worthwhile [58]. 

### 3.3. Thermal Conductivity

Thermal conductivity determines the charge/discharge rate of thermal energy (cooling power). To lower the temperature difference between the charging and discharging phases and hence improve the system’s dynamics, a high thermal conductivity is needed. 

#### 3.3.1. Experimental Measurement

There are several techniques for measuring thermal conductivity, including the transient hot-wire method (THW) [149,150], the 3ω method [151], the combination of the two, the 3ω-wire technique [57], and the laser flash method. Some studies have reported measurements by different thermal analyzer models (hot disk TPS 2500, KD Pro32) that are available commercially. 

The first method, THW, was applied for the first time in 1931 and further developed for electrically conducting fluids [150]. Given the reported charge of nanoemulsion droplets [59], THW was a suitable technique and came to be used by many researchers [57,58]. In this method, the increase in the temperature of a test sample over time when heated by a thin, directly immersed hot wire, is recorded. The short measuring time used (a few seconds) when the temperature variation is small means that there are no significant convection effects and thus ensures reliable measurement, based on the equation:

(24) $$k=\frac{{\dot{q}}_{l}}{4\pi \frac{∆T}{∆(\log t)}}$$

where ${\dot{q}}_{l}$ is the heat flow per unit length and the ratio ∆T/∆(logt) is the slope of the temperature variation ∆T as a function of the log (time) variation, ∆(logt). 

Some of this technique’s major advantages relate to its high accuracy, fast measurement time, and relatively simple experimental setup. 

In the 3ω method, the fluid conductivity is measured by detecting the frequency dependence of the temperature oscillation in a metal wire used as a heater and thermometer [151,152], with reported errors of less than 2%. The method can be applied for a range of fluids as well as for thin films, and the thermal diffusion is calculated from the equation:

(25) $$k=\frac{V^{3}ln\omega_{1}/\omega_{2}}{4\pi lR^{2}\left(V_{1}-V_{2}\right)}\frac{dR}{dT}$$

where $V_{1}$ and $V_{2}$ are, respectively, the in-phase 3ω voltages at frequency $\omega_{1}$ and frequency $\omega_{2}$; *V* is the voltage across the metal line at ω; and *dT/dR* is obtained from the calibration temperatures as a function of the resistance [151]. 

The 3ω-wire method is a combination of the 3ω [151] and THW methods [42,112,153,154]. A metal wire is immersed in the liquid and a sinusoidal current of frequency ω is passed through, generating heat at a frequency of 2ω, which is determined by the voltage component at a frequency of 3ω. The thermal conductivity can be established from the slope of the temperature rise with respect to the frequency ω according to the equation:

(26) $$k=\frac{P}{4\pi l}{\left(\frac{\partial T_{2\omega }}{\partial ln\omega }\right)}^{-1}$$

where *P* is the applied electric power and *l* is the wire length. One proclaimed advantage of the 3ω-wire method is that the temperature oscillation can be more easily kept within lower limits (under 1 K) than that for the THW method; thus, liquid properties remain constant. Additionally, this method seems to be more suitable for measuring temperature-dependent thermal conductivity. 

The laser flash method involves firing a laser pulse and measuring the heat output while simultaneously recording the temperature gradient through the sample thickness. The thermal diffusivity is thus measured and, based on known values of density and specific heat capacity, the material thermal conductivity is determined. This method is usually used to measure solid samples, but it is sometimes used for liquids as well [155]. 

#### 3.3.2. Theoretical Aspects and Validating Data

**Figure 5:**
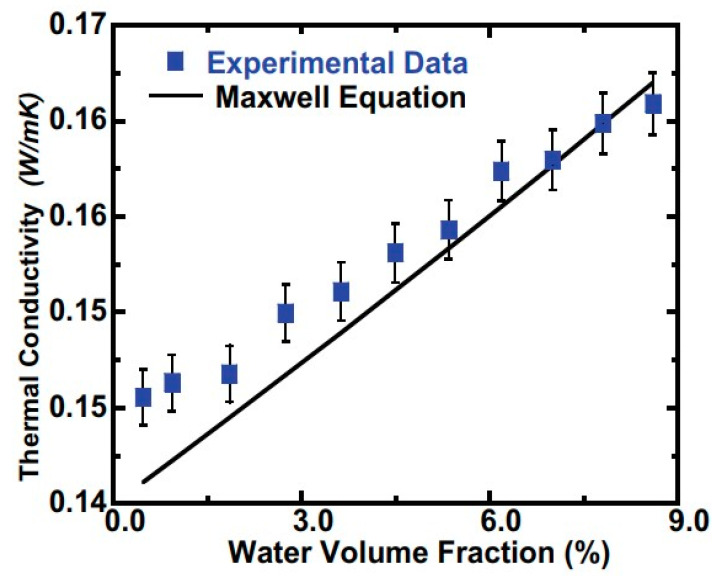
Variation in the thermal conductivity of a water/polyalphaolefin nanoemulsion with the water volume fraction [57] (reproduced with permission).

Maxwell proposed an equation for suspensions of uniformly dispersed, noninteracting spherical particles [156,157] in the form of:

(27) $$k_{M}=\frac{k_{d}+2k_{c}+2\varphi \left(k_{d}-k_{c}\right)}{k_{d}+2k_{c}-\varphi \left(k_{d}-k_{c}\right)}k_{c}$$

where $k_{d}$ is the thermal conductivity of the particles, *k_c_* is the thermal conductivity of the base fluid, and φ is the particle volumetric fraction. The expression indicates an increase in the thermal conductivity with an increase in the particle volumetric fraction, provided the particle shape does not change, and is verified experimentally for dilute nanosuspensions/nanoemulsions with φ<0.1 [57,112]. Using the 3ω-wire method, Xu [57] measured an increasing thermal conductivity for a water/PAO nanoemulsion when increasing the water volume fraction, with a maximum value of 16% observed at a water concentration of 8.6%. The results agreed reasonably well with the Maxwell model (Equation (27)), as seen in Figure 5 [57]. Larger increases of up to six times with respect to the base fluid were reported in other studies [102] for water in an n-decane nanoemulsion stabilized by sorbitane monolurate, with a good fit shown between the experimental data and the Maxwell equation only when the thermal conductivity of the emulgator was accounted for.

Sivapalan [138] reported a continuous decrease in the thermal conductivity of up to 37% with an increase in the paraffin wax concentration in water of up to 50 wt%, even though an increase in the heat capacity was registered. Cabaleiro [30] also reported a decrease in the thermal conductivity of PCME for a paraffin-in-EG+water (RT21HC/EG+W) nanoemulsion and found a good compatibility with the Maxwell model for low volume fraction values, when the droplets were in a solid state. Similar findings were conveyed for n-decane-in-water nanoemulsions [158] and for n-hexadecane-in-water emulsions with a 10–30% dispersed phase concentration [131]. 

It is clear that the Maxwell equation can be used when nanodroplets are not prone to strong deformations, particle–fluid interactions, Brownian motion, and other possible effects, which depend on the diameter, concentration, temperature of dispersed phase droplets. However, when the nanoparticle concentration is increased beyond a certain value—roughly 30%—deviations from the Maxwell equations are usually recorded, which suggests that droplet interactions dominate the Brownian motion, thus changing the underlying physics of the nanoemulsion thermal conductivity evolution.

The apparently conflicting results regarding the increase or decrease in the thermal conductivity with the volume fraction of the dispersed phase are simply due to the high (water) or low (paraffin) thermal conductivity contribution of the dispersed phase to the thermal conductivity of the whole nanoemulsion. 

On the other hand, if the medium anisotropy due to small particle clusters and interface effects needs to be accounted for, the effective medium theory can be used to predict the thermal conductivity of the nanoemulsion. When the dispersed medium is considered to be spherical and the resistance at the boundary between the two media is accounted for, the Maxwell–Garnett equation can be used:

(28) $$k_{eff}=\frac{k_{d}\left(1+\gamma \right)+2k+2\varphi \left[k_{d}\left(1-\gamma \right)-k\right]}{k_{d}\left(1+2\gamma \right)+2k-\varphi \left[k_{d}\left(1-\gamma \right)-k\right]}k$$

where γ is the ratio between the interfacial thermal resistance and particle size. Regardless of the particle conductivity or the volume percentage values, this model predicts an increase in the effective conductivity for γ>1 and a reduction for γ>1 [159]. Therefore, for nanoemulsions it might be more appropriate to compare experimental data to the Maxwell–Garnett equation rather than to the simplified Maxwell model. 

#### 3.3.3. Thermal Conductivity Dependence on Temperature

The Brownian motion of the particles is quantified by the Brownian diffusion coefficient, $D_{B}$, given by the Stokes–Einstein equation:

(29) $$D_{B}=\frac{K_{B}T}{3\pi \mu d_{p}}$$

where $K_{B}$ is the Boltzmann constant, *T* is the absolute temperature, μ is the fluid viscosity, and $d_{p}$ is the particle diameter [160]. This equation indicates an increase in the rate of collisions with an increase in temperature, which represents an increase in the thermal conductivity. This is confirmed by experimental data [58]. Despite the very small sizes of particles (0.8 nm), Xu [73] found slight increases in the thermal conductivity of ethanol in a PAO nanoemulsion with an increase in temperature; thus, no major impacts of Brownian motion on thermal transport were detected experimentally. For a paraffin-based NE (n-hexadecane/water), Chen [58] reported a slight increase in the apparent thermal conductivity, measured using the THW method, when the temperature went beyond the n-hexadecane nanoemulsion phase change temperature, followed by a sharp decrease at the melting temperature when the solid phase (higher thermal conductivity) became liquid (lower thermal conductivity). The thermal conductivity of PCE increased significantly in comparison to the thermal conductivity of pure paraffin, with the highest value being registered at the lowest paraffin concentration. 

Constant thermal conductivity values for water-based nanoemulsions of tricosane were recently established within a low temperature range (20 °C–40 °C), with a significant increase being detected during heating within the phase change temperature interval [141]. 

PCM’s poor thermal conductivity causes a sluggish transmission of heat and low heat storage and release rates, which is a significant disadvantage in practical applications. In latent heat transfer systems, heat transport can be improved by the use of a geometric configuration and/or an improvement in thermal conductivity. Therefore, the introduction of highly thermally conductive metallic nanoparticles [62], carbon-based nanoparticles [161], metallic foams [162,163], expanded graphite, and the encapsulation of PCM can enhance the thermal conductivity [159]. For example, the thermal conductivity of PCM fluids was found to increase by about 10.7% at 30 °C and 12.9% at 90 °C when metallic particles of indium suspended in PAO were used [73]. Another typical strategy for improving heat transmission in latent heat thermal energy systems with a low thermal conductivity includes the use of extended surfaces, such as fins [164] or heat pipes, with reported increases of up to 300% over time after the phase change has occurred. However, these inclusions usually suppress convection, which seriously impacts the melting/thawing phenomena; thus, an optimum placement and orientation of fins inside the system is needed. 

For nanofluids, thermal conductivity increases with the solid particle concentration as well as with temperature, undergoing a ~30% increase in the temperature range of 25–50 °C, for particle concentrations of up to 2.4% [165]. The thermal conductivity increase is also favored by smaller-diameter particles [45] and can be influenced by their shape—for example, a blade-like shape can lead to an increase of up to 60% [44]. 

The reported data for the enhancement of the thermal conductivity of fluids seem to be more significant for nanofluids than for nanoemulsions; therefore, the use of hybrid or composite nanoemulsions [166] may offer a better alternative. 

### 3.4. Specific Heat Capacity and Phase Transition Enthalpy

The nanomaterial specific heat capacity, *c_p_*, is one of the most essential properties in the design of an effective storage system because it directly influences the storage capacity and the ability to increase the amount of heat that may be stored or transferred; thus, knowledge of this is necessary in order to analyze the system energy performance. For a nanoemulsion used to store sensible heat, the heat capacity is given by:

(30) $$c_{NE}=\Phi c_{d}+\left(1-\Phi \right)c_{c}$$

where Φ is the mass fraction of water and $c_{d}$ and $c_{c}$ represent the heat capacities of the dispersed and continuous phases, respectively. In this case, the NE heat capacity can be increased by simply using a dispersed phase with a higher specific heat capacity. 

Another method that can be adopted to increase the overall system heat capacity is to use a phase-changeable dispersed material. The nanoemulsion effective heat capacity can be written in this case as:

(31) $$∆H_{NE}=\Phi \cdot ∆H_{PCM}+\left(1-\Phi \right)\;c_{p}∆T+\Phi \bar{c_{p,PCM}}\;∆T$$

where $∆H_{NE}$ is the total heat capacity of the nanoemulsion (PCE), $∆H_{PCM}$ is the latent heat of the dispersed phase change material (PCM), $c_{p}$ is the heat specific capacity of the base fluid, $\bar{c_{p,PCM}}$ is the mean specific heat capacity of the PCM, Φ is the mass fraction of the dispersed phase, ∆T is the temperature difference, and the emulgator heat capacity is neglected. 

Obviously, the last method is more efficient, since the latent heat has much larger values in comparison to the material heat capacity. 

#### 3.4.1. Experimental Measurements

The most widely used technique to measure *c_p_* is differential scanning calorimetry (DSC), which simply determines this property based on the heat flow difference between a sample and a blank (no sample) test under identical conditions. The heat capacity is determined by the heat flow, the temperature rise, and the sample mass based on the equation:

(32) $$c_{p}=ct.\frac{q_{s}-q_{b}}{∆T}$$

where *ct.* is the calibration constant; $q_{s}$ and $q_{b}$ are the heat flow for the sample and the blank, respectively, measured over a certain time period; and ∆T is the temperature change over the same time interval. 

This method also gives information about the amount of heat stored or released during the phase change based on the sample weight, the heating and cooling rates, and the estimated peak area of the obtained DSC curve. 

The procedure is based on the standard ASTM 1269E [167]. Despite its obvious advantages, the DSC technique uses very small samples of a few mg, which, to some extent, are not representative of the true structural characteristics of NE; thus, it is not known how the real values of the measured property may change. 

Another used technique for *c_p_* measurement is modulated or standardized DSC (MDSC) [168]; this is more accurate than the classical method and uses discrete Fourier transform, where the temperature and heat flow sample amplitudes are compared to those of a reference wave with the same frequency. The heat capacity is calculated based on the following equation:

(33) $$c_{p}=ct.\left(\frac{q_{amp}}{T_{amp}}\right)\frac{\hat{T}}{2\pi }$$

where *ct.* is the calibration constant, $q_{amp}$ is the heat flow amplitude, $T_{amp}$ is the temperature amplitude, and $\hat{T}$ is the modulation period. 

Still, DSC remains the most widely applied method, as it allows for several simultaneous measurements of thermal properties of interest. 

**Figure 6:**
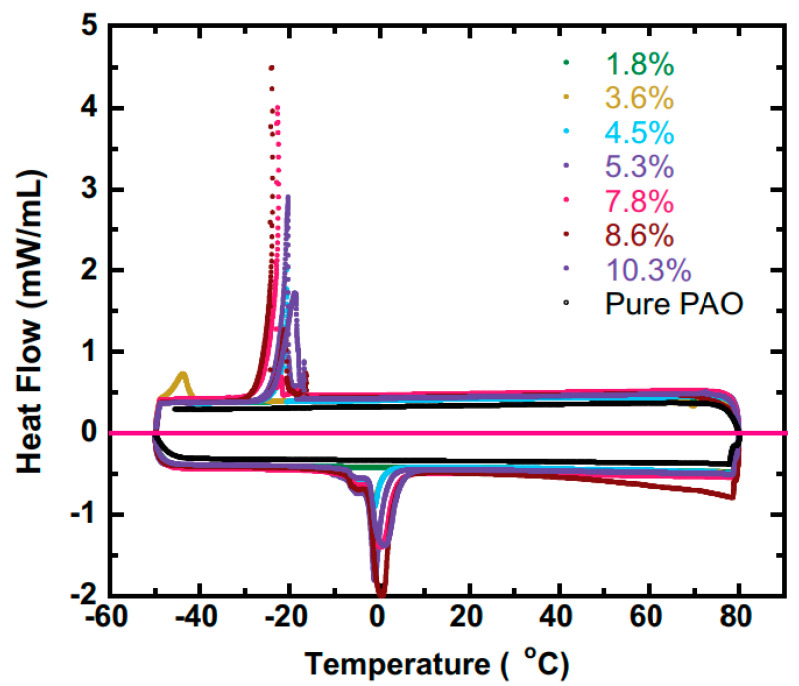
Repeated DSC measurements for the melting latent heat determination of a water-in-polyalphaolefine nanoemulsion [57] (reproduced with permission).

As already mentioned, the enthalpies of phase transitions can be also determined experimentally using the same DSC technique by integrating the area under the peak corresponding to the given transition according to:

(34) ∆H=cA

where *A* is the area under the peak and *c* is the calorimetric constant. Typical DSC signals used to determine the latent heat of transformation are represented in Figure 6. 

DSC plots also facilitate the determination of PCM’s peak temperatures—namely, the melting and solidification temperatures, as well as the onset and end-set temperatures, meaning the temperatures at which melting begins and crystallization ends, thus leading to a supercooling effect. 

#### 3.4.2. Parameters of Influence

The specific heat capacity was found to increase with an increasing mass fraction of dispersed droplets for a nanoemulsion with no phase change. For example, for a water in FC-72 (fluorinate electronic liquid) nanoemulsion with no phase change, the measured heat capacity undergoes an increase of over 15%. This was achieved for a water volumetric fraction of 12%, and this result was also verified by the mixture law (Equation (30)) [73]. Other studies have reported similar enhancements with an increase in the dispersed phase concentration [111]. 

Since the potential for energy storage is much larger if a PCM is used, numerous studies have focused on different phase-changeable heat transfer nanostructured fluids—namely, solid–liquid fluids (e.g., water-in-PAO [57] and paraffin-in-water nanoemulsions [11,58,138,140]) or liquid–vapor fluids (e.g., ethanol in PAO [73]). 

Xu [57] investigated the effective specific heat variation for a water/polyalphaolefine nanoemulsion at different water concentrations. It was found that for a water concentration increase from 4.5% to 5.3 %, the heat of fusion was enhanced from 9.8 J/g to 26.72 J/g, and this continued increasing up to 34.17 J/g, when the concentration reached 8.6%, according to data obtained from Figure 6. This sharp increase of approximately 76% was attributed not only to the phase change, but also to a structural change in the droplets from a spherical shape to a cylindrical configuration, which was confirmed by small-angle neutron scattering (SANS) measurements. The favorable alterations in the nanoemulsion dispersed phase structure can be attained in other systems, as well, since a significant increase in heat capacity can be obtained. 

Almost linear increasing trends for heat capacity were also reported for paraffin wax–water/Pluronics P-123 nanoemulsions obtained via ultrasonication when the paraffin wax concentration was increased; the highest value of 43% was registered at a wax concentration of 33% [138]. 

Xu [73] reported increases in the specific heat of water of more than 200% in FC-72 nanoemulsion fluids at the liquid–solid phase transition for a 12% volume fraction of water. 

Chen [58] also found that the latent heat of the PCE at different paraffin mass fractions agreed well with theoretical predictions (see Equation (31)), while the total heat capacity of the NE increased about 50% with respect to the water heat capacity for a concentration of n-hexadecane of 10% as well as for n-octadecane in water nanoemulsions. Another interesting finding was related to the decrease in both the latent heat and the melting temperature of water in PCE in comparison to that of pure water, an effect that became even milder with the increase in the mass fraction of the PCE. This was explained based on the presence of the surfactant by investigating the latent heat of a water–surfactant mixture with the same emulgator concentration as the PCE. The mixture latent heat presented deviations similar to those of the PCE; however, these were larger than those for the PCE, which was explained based on the smaller number of micelles formed in the PCE (larger droplets) and by the decrease in the van der Waals forces acting between the water molecules inside the PCE. 

One of the reported problems during the phase change in PCMs is related to the large melting–freezing hysteresis thought to occur due to differences in the nanodroplet interface free energy and their diameters [58,111]. The presence of a single peak implies that the phases are well-dispersed and that the nanodroplets are almost the same size; therefore, the DSC technique can provide qualitative information on the NE polydispersion and stability. Another observed effect during the phase change period is supercooling. The PCM does not solidify immediately after the melting temperature is achieved but rather has to be cooled below the solidification temperature in order to start crystallizing and release latent energy. This is defined as the difference between the onset temperature of the melting, *T_mt_*, and the end-set temperature of the freezing process, *T_fr_*:

(35) $$∆T=T_{mt}-T_{fr}$$

Therefore, supercooling defines a temperature range that must be applied to realize the entire potential of PCM [19,169]. The wider this range is, the lesser the benefit of the stored energy will be. Several studies have reported the use of nucleating agents in PCME [30] or in other phase change material systems [170] in order to promote nucleation. However, for nanoemulsions, partial reductions in this effect (up to few degrees) were achieved and only slightly better heat storage capacities were attained [30]. Thus, supercooling and crystallization rate become important parameters for the design of a thermal storage system and require more detailed investigations related to the kinetics of nucleation within a PCME and the parameters that influence it. 

Regarding the variation in the specific heat of nanofluids, this was found to decrease by as much as 25% with a particle concentration increase within 5% to 25% [171], which clearly indicates that phase change nanoemulsions represent a better option for thermal storage. 

### 3.5. Surface Tension

Surface tension is the tendency of a liquid surface to shrink in order to attain the minimum surface area. Since it relates to the cohesive forces within the system and represents the surface energy per unit area, it controls the droplet shape, which can influence the heat performance of a system [172]. The surface tension can affect the heat transfer because colloidal particles can accumulate at the interface and change the interfacial tension of the fluid, affecting its wetting properties and leading to a change in the transported thermal energy. Surface tension plays an important role in the formation of droplets, in the boiling film drying, and in the critical heat flux value. 

#### Experimental Measurement

Interfacial tension and surface tension can be measured using different techniques, such as the DuNoüy ring method, Wilhelmy plate, spinning drop, and pendant drop [173]. The latter is often used due to its versatility and robustness. It consists of fitting a Laplacian curve, expressed by Equation (36), to an experimentally recorded profile and minimizing the errors involved [174]:

(36) $$\sigma \left(\frac{1}{r_{1}}+\frac{1}{r_{2}}\right)=∆p$$

Computational routines are utilized to greatly increase the precision of the method. In this case, different drop shape analyzers (DSA–30 from Krüss GmbH) [30] can be used. 

Contact angle measurements use the sessile drop method, which consists of placing a drop of liquid on a solid plate submerged below the surface of another liquid. A photograph of the system is taken in order to obtain an accurate measurement of the contact angle [175]. 

All the above specified measurements are usually conducted in an environmental chamber in order to control or study the dependence on temperature. 

According to experimental measurements, the surface tensions of PCMEs were found to decrease by up to 50% when paraffin droplets were present; thus, they were smaller than those for the water–emulgator (sodium dodecyl sulphate) mixture employed to make the nanoemulsions [30]. 

Similarly, for the paraffin-in-water NE, the contact angles were up to 52% lower than those for water; however, the samples maintained their shapes on a stainless-steel surface in contrast to the paraffin or emulgator–water mixtures [30]. Despite the reported decrease in surface tension, which would intensify the heat transfer process, the use of certain surfactants, such as polyethylene glycol 400 and sodium alginate, may have negative effects on the stability of PCMs [176]. Systematic studies regarding the impact of surface tension on heat transfer during the charging/discharging cycle have not been performed, so little is known about the impact of this thermal property on the operation of TES systems. 

For nanofluids, there are conflicting reports regarding the variation in surface tension with nanoparticle concentration [177,178]. At the same time, decreases in surface tension with increases in temperature and the addition of a surfactant have also been reported [176]. 

For nanoemulsions, all thermo-physical properties relevant for thermal storage and heat applications, including their variation with droplet concentration and temperature, are summarized in Table 3.

### 3.6. Other Measurements 

#### 3.6.1. Thermal Measurements

##### T-History Method

**Figure 7:**
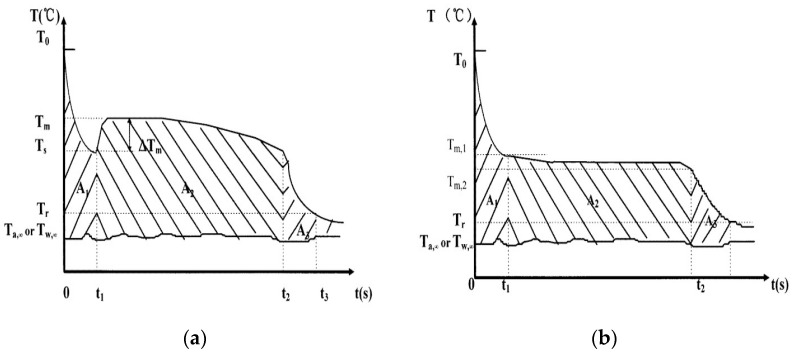
Typical PCM response during a cooling process (**a**) with supercooling and (**b**) with no supercooling [92] (reproduced with permission).

**Figure 8:**
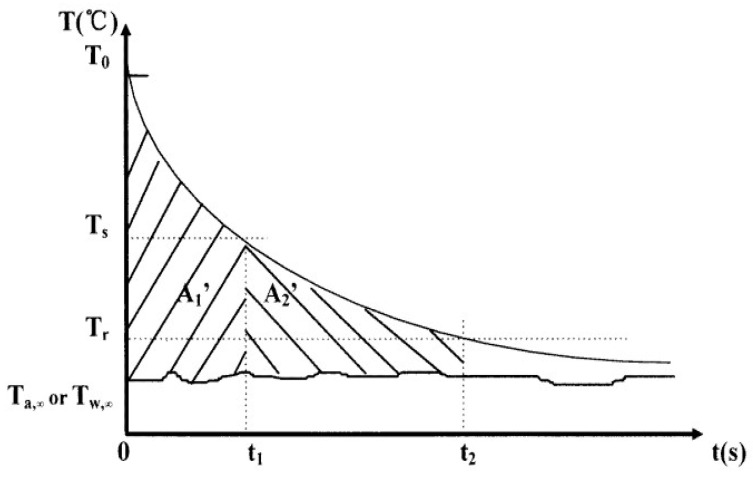
Typical T-history curve for water cooling [92] (reproduced with permission).

Another method that can be used to simultaneously determine, within a laboratory simple setup, the specific heat capacity, thermal conductivity, heat of fusion, and melting temperature is called the T-history method, which was proposed by Zhang [92]. The sample was placed into a tube with a large H/D ratio at a temperature *T_o_ > T_m_*, which is the melting temperature. It was then immersed in a bath at a temperature $T_{a,\infty }$ in a time-dependent manner. The sample temperature was recorded over time, such that, depending on the degree of supercooling (whether it was different or equal to 0), graphs similar to the ones represented in Figure 7a,b were obtained. Additionally, a reference water sample was used to obtain similar temperature–time data, as presented in Figure 8: 

Based on the lumped capacitance method (Bi < 0.1, Bi is the Biot number), the equations for equal heat quantities either stored or exchanged through convection were written together with corresponding equalities for the water system, and the expressions (37)–(40) were proposed to calculate the following thermal properties:

(37) $$c_{p,s}=\frac{m_{w}c_{p,W}+m_{t}c_{p,t}}{m_{p}}\frac{A_{3}}{A_{2}^{\prime }}-\frac{m_{t}}{m_{pr}}c_{p,t}$$

(38) $$c_{p,l}=\frac{m_{w}c_{p,W}+m_{t}c_{p,t}}{m_{p}}\frac{A_{1}}{A_{1}^{\prime }}-\frac{m_{t}}{m_{pr}}c_{p,t}$$

(39) $$H_{m}=\frac{m_{w}c_{p,W}+m_{t}c_{p,t}}{m_{t}}\frac{A_{2}}{A_{1}^{\prime }}\left(T_{0}-T_{s}\right)$$

(40) $$H_{m}=\frac{m_{w}c_{p,W}+m_{t}c_{p,t}}{m_{t}}\frac{A_{2}}{A_{1}^{\prime }}\left(T_{0}-T_{m,1}\right)-\frac{m_{t}c_{pt}\left(T_{m,1}-T_{m2}\right)}{m_{pr}}$$

Equation (39) is used for cases with no subcooling and Equation (40) is used for subcooled systems. In these, $m_{W}$, $m_{t}$, and $m_{pr}$ are the mass of the water, tube, and probe, respectively; $c_{p,s}$, $c_{p,l}$, $c_{p,W}$, and $c_{p,t}$ are the specific heat capacities of the solid and liquid samples, water, and tube material, respectively; and *A_1_, A_2_, A_3_,* $A_{1}^{\prime }$*,* and $A_{2}^{\prime }$ are the integrated areas under the temperature–time curves presented in Figure 7 and Figure 8. 

This method was used to measure several salt hydrates, paraffin, and some other PCM systems. Comparisons between the obtained experimental data and other literature results showed a reasonably good agreement. The major advantages of the T-history method relate to the simplicity of the experimental setup, the ability to achieve the simultaneous collection of several thermo-physical properties within the same run, and the possibility of running the test on several samples at the same time. Additionally, the investigated temperature value interval depends on the type of bath used (water or oil), which makes it versatile for many different PCMs as well as allowing larger samples to be tested than would be possible in the DSC method, making it more relevant for the particle distributions and interactions occurring inside the NE. Despite possible errors related to the experimental measurement, this method is extremely helpful, especially for the thermal characterization of newly designed phase change nanomaterials. 

Recently, the T-history method was successfully applied for microencapsulated phase change material slurry concentrates, where a water solution of propylene glycol is used as a base liquid [179].

###### Thermal Gravity Analysis (TGA) 

Thermal gravity analysis indicates the temperature at which the PCM decomposes; thus, it can be used to verify if the working temperature interval for charging/discharging cycles is far from the value measured, such that the system can be used repeatedly. For example, Veerakumar [180] determined the decomposition temperature for a capric acid/cetyl alcohol binary eutectic phase change material used for cold thermal energy storage, concluding that it can be safely used. 

#### 3.6.2. Measurement Techniques for Structure Identification

##### Light Scattering Methods

Light scattering is a well-established nonintrusive technique for determining the size, molecular weight, diffusion, and interaction strength of particles in solutions, with notable advantages including quick measurement times, high precision, and lack of pre-calibration requirements. Dynamic Light Scattering (DLS) has been intensively used to determine the sizes of droplets inside nanoemulsions. The intensity–time fluctuations of reflected or transmitted light beams can be measured after the sample is treated with a light beam. The frequency shift of the incident light beam when dispersed by particles in motion inside a colloidal suspension can be used to calculate the particle sizes. The obtained data are the intensity-weighted mean of the hydrodynamic diameter and the polydispersity index (PDI), which represents the width of the particle size distribution and, as a result, the homogeneity of the formulation. A PDI of less than 0.2 indicates a narrow droplet size distribution, while a value close to unity shows heterogeneity. Furthermore, commercially available DLS equipment allows for measurements of particle charge or zeta potential, which are important parameters indicating the stability of a nanoemulsion. A value of ±30 mV is thought to be sufficient for obtaining a high NE stability over time [39,181]. 

##### Small-Angle Neutron Scattering (SANS) 

The size of nanoemulsion droplets can be also established in situ by using small-angle neutron scattering [86] and small-angle X-ray scattering (SAXS). The main advantage of this method is related to measurements being performed on colloidal suspensions of higher concentrations (>1%vol.) [57], unlike in traditional DLS. 

##### Nuclear Magnetic Resonance Spectroscopy

NMR is a technique used for analyzing concentrated, opaque nanoemulsions, for which sample dilution is not required. The solid content of a sample is determined by measuring the interactions of radio waves with the nuclei of hydrogen atoms in the sample. The amplitude and decay time of the NMR signal produced by the hydrogen nuclei’s excitation state is measured to determine the ratio of solid to liquid droplets in a nanoemulsion. 

##### X-ray Diffraction

Another powerful approach for tracking chemical deterioration while a material is being processed or tested is X-ray diffraction. This technique involves diffracting X-rays when they are applied to a sample. Each signal obtained is unique to the material, and if a degradation process is taking place, the diffractogram will alter dramatically. The XRD technique has been successfully utilized [10] to track PCM degradation. 

##### Infrared Spectroscopy (FTIR) 

Infrared spectroscopy (FTIR) is one of the most widely used and adaptable techniques for monitoring chemical degradation following a treatment. It is commonly employed to confirm structural chemical changes in various systems. The signal obtained is a characteristic peak that may be identified as corresponding to a single chemical compound (or a family of substances). The vanishing or reappearance of typical peaks indicates that a material is degrading. In the case of PCM, the investigation method was used to assess the stable chemical structure of a eutectic based on capric acid/cetyl alcohol for cold thermal storage after 1000 thermal cycles, with no noticeable structural variations [169], which basically qualified it as a suitable PCM system. 

## 4. Integrated Thermal Properties Relevant for TES Design and Operation

Thus far, the above discussed thermo-physical properties are recognized to have a direct effect on the characteristics of nanoemulsions, the pumping power required for their transportation, and their capacity to store energy. No matter how much these properties vary, the performance of TES fluids should be defined by a combination of them rather than by their separate values. Some studies have proposed the use of non-dimensional expressions comprising grouped thermophysical properties—for example, a figure of merit, which is established based on different approaches to the heat transfer within a system or dimensionless numbers, which are characteristic of the thermal energy transport mechanisms occurring in heat storage systems. 

### 4.1. FOM Expressions

The figure of merit comprises thermo-physical properties that are weighted through exponential coefficients in order to assess their contribution to the heat transfer process; it can be used to compare different TES materials. For example, based on the Dittus–Boelter correlation, an adimensional number—the Mouromtseff number [182]—was proposed in order to account for the influence of fluid properties on the convective heat transfer coefficient:

(41) $$Mo=\frac{\rho^{0.8}c_{p}^{0.33}k^{0.67}}{{µ}^{0.47}}$$

Considering the forced turbulent convection inside a heated collector tube, the figure of merit established by Equation (42) [183] facilitated the creation of a hierarchy of some materials of interest in terms of their heat transfer efficiency as follows: liquid metals, molten salts, oils, and gases.

(42) $$FOM=\frac{\rho^{2}c_{p}^{1.6}k^{1.8}}{\mu^{1.4}}$$

However, these figures of merit do not account for thermal and hydraulic performances when the carrier fluid undergoes a phase change within the system. To consider this, Shamberger [184] proposed another FOM starting from the analytical solution of the two-phase Neumann–Stefan problem of the melting of a semi-infinite material with fixed boundary conditions; Yang [185] used it in the form of the effective cooling capacity:

(43) $$\eta_{eff}=\sqrt{k_{eff}{∆H}_{,\;eff}}$$

Additionally, based on its values for different PCM-containing conductors, he discussed their decreasing efficiency in thermal storage. 

Despite the fact that these figures of merit have been proposed based on several well-known correlations, none of them includes all the requirements necessary for the most efficient transport, storage, and handling of thermal energy. A detailed comparison between different PCMs could be performed once a more general FOM is found. 

### 4.2. Dimensionless Numbers

The practical implementation of a PCM should include the proper investigation of the material behavior in the thermal facility and the predictable operation of the whole system, both as a sink and a source of energy. Therefore, it is probably more appropriate to discuss criteria that include these properties and that can also provide more information about the interactions of the storage system with the environment. 

For example, when a material is heated its thermal diffusivity, α, is given by:

(44) $$\alpha =\frac{\rho c_{p}}{k}$$

This indicates how well a material can disperse heat, taking into consideration both how quickly heat can be transferred through it (thermal conductivity) and how quickly its own temperature can change (heat capacity). The faster the temperature varies and the greater α is, the higher the penetration distance will be and the higher the speed with which the temperature adapts to the thermal variations in the environment will be. α does not dictate the size of a heat flow. On the other hand, thermal effusivity, also known as thermal permeability, ε, is given by:

(45) $$\varepsilon =\sqrt{\rho c_{p}k}$$

This dictates the ability of the system to exchange thermal energy with the outside. Materials with a high ε change large amounts of energy if there is enough driving force—i.e., temperature difference. However, the material internal temperature distribution is still dictated by the thermal diffusivity value. 

However, these observations limit the discussion to a system where heat conduction is the only occurring heat transfer mechanism—namely, to a solid-state PCM. For a PCM slurry, the convection heat transfer mechanism becomes significant and affects the temperature distribution and the rate of transfer, with the whole process being of a transient nature. 

Several nondimensional numbers can be used to characterize such a system—for example, the Stefan number, defined as the ratio between the sensible heat stored in the material over a defined temperature range and the latent heat of fusion ∆H when the solid–liquid phase change occurs:

(46) $$St=\frac{c_{p}∆T}{∆H}$$

For this, the temperature range ∆T needs to be carefully defined, since it may have the same value for PCM systems that warm up and melt differently or exchange heat at a different rate [186]. The Stefan number can also be used in a modified form to quantify the subcooling effect [187]. Thus, for PCM slurries a modified Stefan number reflecting the effect of phase change on heat transfer was proposed [188]:

(47) $$St=\frac{C_{b}\left(t_{fl}-t_{e}\right)-∆H}{∆H}=\frac{q_{w}}{\dot{m}∆H}-1$$

where *q_w_* is the wall heat flux and $t_{fl0}$ and $t_{e}$ are the bulk fluid temperature and the entrance temperature. 

Several experimental and numerical studies underline the importance of the Stefan number in heat transfer in a PCM nanoemulsion. For example, Roy [189] numerically investigated turbulent heat transfer, allowing for the phase change effect in the energy equation, and found that for high heat fluxes, the Stefan number influences the tube length over which the phase change effect is important. The proposed model was verified by experimental data from the literature. It is worth mentioning that similar findings related to the Stefan number as a major parameter, indicating the influence of the phase change on the heat transfer, were also confirmed for microencapsulated phase change materials both numerically and experimentally [190]. 

It is expected that the Stefan number cannot define the PCM behavior by itself, considering that for a regular fluid undergoing heat transfer, several nondimensional numbers can be established based on the differential energy equation sets in a nondimensional form, such as the Grashof number, which defines natural convection; the Reynolds number, which is for forced convection; the Prandtl number, which is a property criteria; and the Nusselt number, which includes the heat transfer coefficient, *h*, and defines how much heat is conveyed by convection with respect to that transferred by conduction. The definition equations for these are as follows:

(48) $$Gr=\frac{\rho^{2}gl^{3}\beta ∆T}{\mu^{2}}$$

(49) $$Re=\frac{\rho vl}{\mu }$$

(50) $$Pr=\frac{\rho c_{p}}{\mu }$$

(51) $$Nu=\frac{hl}{k}$$

where *β* is the volumetric thermal expansion coefficient; *ρ*, *µ*, *k*, and *c_p_* are the fluid density, viscosity, thermal conductivity, and heat specific capacity, respectively; *g* is the gravitational acceleration; and *l* is a characteristic length depending on the device geometry. These criteria can be extended for PCMEs and clearly are important for quantifying the ongoing heat transfer process. Furthermore, some studies propose the use of the Fourier number, as in:

(52) $$Fo=\frac{\alpha t}{l^{2}}$$

This is a dimensionless number that considers the time, t, required to arrive at a given cold heat storage state for a nanoemulsion [191]. 

Several studies have been dedicated to the investigation of the flow and heat transfer characteristics of PCEs in channels [192] and circular/annular tubes [140,192] in either laminar or turbulent flows; the results indicated that the PCEs exhibited significant heat transfer enhancements of approximately 15% to 45% under turbulent forced-flow conditions, depending on the Re value. The heat transfer coefficients were found to increase significantly close to the phase change temperature [192]. Additionally, for paraffin-in-water nanoemulsions with relatively low concentrations of the dispersed phase, ≤ 10%, standard heat transfer correlations rendered heat transfer coefficients close to experimentally measured values [139]. Experimentally verified, classical heat transfer correlations for Nusselt calculations, such as Sieder–Tate (SD), Ditus–Boelter (DB), and the more general Gnielinski (G), are presented in Table 4 for different PCME systems. Please note that some of the included equations pertain to microencapsulated phase change materials (MPCM), since the similarities with solid–liquid or even liquid–liquid nanoemulsions are obvious. 

Interestingly enough, for laminar flow, a theoretical study supported by experimental data showed that significant heat enhancements can be obtained due to microconvection induced by dispersed particle rotation in shear flow, an effect that is modeled by the shear-dependent thermal conductivity [198]. Additionally, for a laminar regime with a phase change, Chen [196] reported an increase in the heat transfer coefficient in a laminar phase-change slurry flow in the form of Equation (60), which underlines the significance of the contribution of latent heat to the increase in the heat transfer coefficient. 

Additionally, a comparison of Equations (61) and (62), from Table 4, which have a negative and positive power, respectively, of the Stefan number highlights its stronger impact on the heat transfer coefficient in a turbulent flow than in a laminar flow. Additionally, the direct influence of Re and Pr numbers, and thus of convection, on heat transfer coefficients demonstrates the contribution of convection to heat transfer over conduction. 

Additionally, in the case of turbulent PCME flows, one can also speculate regarding heat transfer intensification as a result of a boundary layer thickness reduction when nanosized droplets flow right near the wall, generating additional turbulent eddies and causing microconvection, since this is known to be a way to intensify property transport. 

Many studies report a PCM heat transfer enhancement of 30% up to 200% in certain Re ranges or even a lower heat transfer coefficient than that for the base fluid in certain working conditions. Some calculations rely on a constant Re number, meaning that the effect of increased viscosity is sometimes not accounted for. The viscosity can typically increase by a couple of times for PCEs, while thermal conductivity has low values (0.2–0.5 W/mK) and can hardly be increased; thus, the heat transfer performance reported in situations of forced convection may be worse than that reported for pure base fluids. Nevertheless, if the heat capacity of the PCE is large enough for a specified heat storage capacity, a smaller mass flow rate will transport the same amount of heat and thus lead to a much smaller pumping power consumption than that of the base fluid [58]. 

Other laboratory setups using PCMs as working fluids and reporting on their energy storage performance include mini-channels [199,200], with a reported increase of 70% in heat transfer coefficient for a water-in-PAO nanoemulsion, and tanks with a helical coil heat exchanger, which have a 34% [200] or even 50% [201] enhancement in energy storage, with the latter value reported for a n-hexadecane/water nanoemulsion. Coiled double-tube heat exchangers [191] have remarkably larger registered values for the Nusselt number and often show an increase in storage capacity by 50–160% in comparison to the base fluid. Shell-and-tube heat exchangers [202] show a 70% increase in the heat transfer coefficient during charging, while plate heat exchangers have shown an enhanced performance compared to conventional storage systems, with an increase in effectiveness of up to 83.1% being reported even when a PCM with a low thermal conductivity was used [203]. Other designs/testing rigs have also been used [204]. On one hand, the above studies indicate the need to possess detailed information about the thermo-physical properties of PCEs as well as the phenomena taking place inside the PCM enclosure, which plays a significant role in thermal storage system design and operation. Regardless of the improved experimental values for heat transfer coefficients and increased energy storage densities, many studies have reported the necessity of the use of new designs in heat exchangers in order to further improve the heat transfer rate and store and release energy more efficiently.

## 5. Discussions

The design and exploitation of the equipment required for the preparation, processing, storage, and transport of the nanoemulsions used for thermal energy packing or other applications necessitates a good knowledge of relevant thermophysical properties, such as density, viscosity, thermal conductivity, thermal capacity, and surface tension. 

A large number of studies have focused on the measurement of thermal properties via different techniques; however, in certain cases these data indicate opposite trends of variation with the concentration of droplets depending on the type of substance used. These differences may originate from distinctive sources, including the method applied for preparation; differences in the geometry, dispersion, and/or interactions of droplets; and the size of the samples and whether these are relevant to the bulk properties. Thus far, many preparation methods have been employed, and it seems that either low-energy or high-energy methods, including ultrasonication and microfluidization, are preferred. A comparative study on different preparation techniques for flavored nanoemulsions revealed their good stability, with droplet diameters of ~100 nm, when heating, microfluidization, or ultrasonication were applied versus hand blending [205]. Another analysis revealed no major differences between the diameters of the nanoemulsion droplets obtained using low-energy techniques—namely, spontaneous emulsification and phase inversion temperature (PIT)—for an oil-in-water nanoemulsion (Labrafil M1944CS/ultrapure water/Solutol HS15) [206]. However, specific substances were used, and therefore the conclusions cannot be extrapolated to other materials or even methods. For thermal storage nanoemulsions, so far, no study has raised the issue of suitable preparation methods in terms of nanoemulsion stability, quality, and related costs. Regarding the influence of sample size on several properties, quantifying this effect may not be as complicated as it seems, since statistical sampling from prepared batches of nanoemulsions and required analyses may clear up this issue. 

Effects of thermal properties on different operational characteristics of a TES system should be integrated depending on the storage space geometry and the enhancement mechanisms responsible for the system’s behavior as a heat source/sink. Important parameters of influence and the corresponding mechanisms of enhancement are listed in Table 5. 

**Figure 9:**
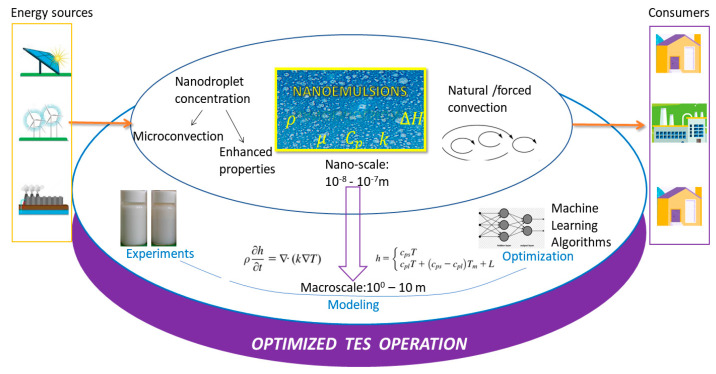
Integration of nanoscale fluids into macroscale TES systems.

Many of the experimental approaches used in studying TES systems follow the same pattern—namely, the preparation of a new nanomaterial followed by property investigation in order to assess some required values for its validation as a TES material, with no further investigation of the thermal efficiency in a given experimental setup. Some studies include certain heat transfer measurements—for example, the heat transfer coefficient or heat transfer rate in a well-known designed setup—in order to evaluate the heat transfer improvement in comparison with that for the pure fluid. However, despite the progress reported, some inefficient use of the PCM flowing space may also be reported. Furthermore, the transient nature of the heat transfer when using phase change materials and the manner in which it influences the rate and efficiency of the transfer have been overlooked. The thermo-physical properties, characteristic dimensionless numbers, and new extended criterial correlations should be investigated in order to better characterize the behavior of TES systems during repeated cycles of charging/discharging. Critical comparative analyses of different heat transfer equipment used for nanoemulsions or other TES materials are also necessary. As already mentioned, the non-steady state nature of these materials is not easy to address. Still, a very effective way to manage this is through numerical simulations and the extensive modeling of the process based on experimental data. Modeling based on supervised learning algorithms and optimization relying on the best learned models can be also an effective way to draw pertinent conclusions. Thus, a full investigation of a new nanomaterial should include the complete characterization of its physical properties in correlation with its structural characteristics. This should also include an analysis of its behavior and performance within a given designed geometry during repeated cycles through corroborating experimental and numerical data, in order to optimize the operation of the TES system that uses it, as well as through neural networks and applied vector machines [210,211,212], as shown in Figure 9. 

Other PCM innovation demands should focus on enhancing the structural stability, improving the stability of thermal cycle characteristics, and reducing corrosion. Additionally, an acceptable system lifespan should be ensured and the performance of the system cycling and the entire system evolution throughout time should be thoroughly analyzed prior to the system’s marketing. All these are essential steps that need to be taken from the laboratory level up to the commissioning of a TES system. 

## 6. Conclusions

Several physico-thermal properties known to directly influence the efficiency of a thermal storage system, such as density, viscosity, thermal conductivity, specific heat capacity, latent heat, and surface tension, have been reviewed in different experimental and theoretical studies, as they are relevant for nanoemulsions with potential use as thermal storage materials. Important issues relating to the heat transfer in such systems have been discussed in order to emphasize the need for a more attentive and unified consideration of properties through dimensionless numbers, aiming to characterize the transfer mechanisms that control key operation parameters such as the rate of transfer, the temperature distribution, and the stored heat density.

Proposing a particular nanoemulsion, such as TES fluid, for use in a functional heat transfer system is challenging, as many factors can affect the final operation of the system. These may be related to the physicochemical features of the constituents, including the oil or aqueous phases; the emulsifiers that play a role in the formation, stabilization, and resulting properties of nanoemulsions; and the geometrical characteristics of the enclosure and heat transfer features, depending on the mechanisms occurring. General algorithms in such cases are difficult to elaborate, since the nanoemulsion type, enclosure size, temperature working interval, and expected time period of operation can seriously modify the variables of importance and, thus, the recommended solutions. Summarizing this viewpoint, several conclusions can be drawn:

- Generally, measurement techniques used for different physical properties render data reliable. However, measurements taken from very small samples may not be able to capture the real compositional characteristics of the material. Comparative studies are needed in order to establish whether this influences the measured values.
- The existing theoretical models need to be further validated by a larger number of experimental data. These are much needed for further numerical and computational modeling and optimization.
- It is known that thermal conductivity determines the charge/discharge of thermal energy (cooling power). Its dependence on the nanodroplet concentration is influenced by the thermal conductivity values of the dispersed phase; thus, it can increase or decrease. However, these variations are not significant. Thus, low thermal conductivity values for nanoemulsions can be further improved using geometrical additions and/or nanoparticles or other composite materials. An increase in temperature induces an increase in thermal conductivity; however, for TES nanoemulsions, charging/discharging cycles imply thermal conductivity variation. Additionally, practical applications may require an effective thermal conductivity, which accounts for the microconvection generated by the nanodroplets within nanoemulsions.
- Latent heat or effective heat capacity governs the energy density of a system. It increases with an increase in the dispersed phase concentration and rises significantly when a phase change occurs. Its contribution to the PCM system is best represented by the use of the Stefan number. However, new correlations, especially for turbulent regimes without and with phase changes and for different geometries, should be proposed.
- Supercooling expands the temperature range that must be used to fully employ a system’s capabilities; therefore, whether this can be reduced or simply used in the future to the consumer’s benefit has to be established.
- Generally, a nanoemulsion’s density increases when the dispersed phase concentration increases and decreases when the temperature increases. However, data related to phase change nanoemulsions have not been consistently reported on, despite their relevance for the TES system, within a working temperature interval.
- While a nanoemulsion’s density does have an impact on thermal storage capacity, its influence on a system’s natural convection and operation may be much more relevant than the value/variation by itself.
- Several theoretical correlations for viscosity that are valid for diluted or moderately concentrated nanoemulsions with rigid spherical or deformable nanodroplets were reviewed. The impact of the solvation effect on a nanoemulsion’s relative viscosity has been verified experimentally for several emulsions; however, further validation would still be appropriate. A new model based on theoretical thermodynamics, classical mechanics flow, and nanoparticle interactions was verified experimentally by several sets of data. These types of models are extremely useful and are expected to provide reliable data, since they have a theoretical basis. The viscosity of nanoemulsions was found to increase significantly at droplet concentrations of 10%; thus, the impact on transport is expected to increase. Some studies advocate for lower velocity transport and thus a reasonable power consumption if the system’s heat capacity is high. Still, an increased viscosity is assumed to have a negative influence on natural and/or forced convection, decreasing the Grashof number (Gr~µ^−2^) and the Reynolds number (Re~µ^−1^).
- A material’s thermal diffusivity affects the depth of heat penetration and the rate of temperature adaptation to a changing thermal environment. Thermal diffusivity determines the system’s thermal inertia, which, in turn, determines the performance of the TES system and its adjustment to user requirements in terms of the power and temperatures imposed. The thermal energy flow is unaffected by the thermal diffusivity. Conversely, the ability of a material to exchange thermal energy with its surroundings, on the other hand, is influenced by thermal effusivity. When there are large temperature differences, the energy flux will be high in materials with a high thermal effusivity. Additionally, natural and/or forced convection seem to have a much more significant contribution to the rate and amount of heat exchanged according to heat transfer correlations. Their individual contributions can be established through numerical analysis and modeling.
- Since the Nusselt number is reported to increase with the concentration of droplets and is known to enhance the heat transfer and most correlations are valid for low concentrations (*φ* < 0.3), new correlations need to be proposed. Additionally, these equations need to include dimensionless numbers relevant for the convection taking place within the system and some characteristic properties and/or characteristic lengths, depending on the geometry of the enclosure.
- For certain nanoemulsions, property-enhancing methods do not render large expected changes. In this area, there is still the opportunity for new discoveries and improvements to be made, most probably based on hybrid or composite nanoemulsions with characteristics better tailored to the target application.
- The convective heat transfer from the wall to the fluid, the hydraulic performance described by the pumping power, and the heat accompanying the phase change occurring in the material determine the performance of thermal nanoemulsions. The expressions proposed for figures of merit account for some of these phenomena by containing properties with different exponential values, allowing for comparisons to be made between different TES materials; however, they do not have a sufficiently high degree of generality (such as nondimensional numbers derived from differential equation of energy).
- The particle geometry of PCME (e.g., cylinders, spheres), the presence and extent of a two-phase mushy zone at the droplet solid–liquid interface, temperature, the droplet concentration dependence of the thermophysical material properties of both the solid and liquid phases, boundary condition assumptions, the geometry of the nanoemulsion’s storage, and the mode of operation strongly influence the system efficiency of TES.
- There is an urgent need to model, optimize, and control the phenomena that take place in such a system in order to attain improved operation with maximum benefits at a reasonable cost.

The measurement and calculation of the physical properties of TES nanoemulsions, which are strongly related to their nano-structural characteristics, are extremely important. However, these must also be correlated with the macro-features of the TES system and integrated to ensure its operation at optimal parameters according to the application requirements for which it was designed.

## Figures and Tables

**Table 1:** Viscosity correlations of nanoemulsions/nanosuspensions depending on the dispersed particle volume fraction, φ.

NE/NS Type	Formula		Observations	Author
Infinitely diluted suspensions, non-Brownian hard spheres	$\mu_{r}=1+2.5\;\varphi$	(4)	For *φ* ≤ 0.03	[114]
Highly diluted suspensions	$\mu_{susp}=\mu_{c}\left[1+\frac{2+5\lambda }{2\left(1+\lambda \right)}\varphi \right]$	(5)	Homogeneous incompressible newtonian fluid	[115]
Highly diluted suspensions of solid particles	$\mu_{susp}=\mu_{c}\left(1+2.5\varphi \right)$	(6)	For *φ* < 0.02	[114]
Moderately concentrated suspensions	$\mu_{r}=\frac{1}{1-K\varphi }$	(7)	*K* = 2 ÷ 2.5(Hess), *K* = 2.5(Ford)	[116,117]
Moderately concentrated suspensions	$\mu_{r}=\frac{1+1.5\varphi }{1-\varphi }$ where μr=μμc	(8)	Accounts for particle interactions, but not for packing; Underestimations for higher concentrations.	[118]
Diluted suspensions	$\mu_{r}=1+2.5\varphi +5.2\varphi^{2}$	(9)	Non-Brownian motion, particle interactions, φ<0.15	[119]
Diluted suspensions	$\mu_{r}=1+2.5\varphi +6.2\varphi^{2}$	(10)	Brownian motion	[120]
Moderately concentrated suspensions	$\mu_{r}={\left(1-\varphi \right)}^{-2.5}$	(11)	For *φ* < 0.02	[121,122]
Higher concentration suspensions	$\mu_{r}=\exp\left(\frac{2.5\varphi }{1-\frac{\varphi }{\varphi_{m}}}\right)$	(12)	For *φ* < *φ_m_*, 0.52 < *φ_m_* < 0.74	[123]
Higher concentration suspensions	$\mu_{r}={\left(1-\frac{\varphi }{\varphi_{m}}\right)}^{-2.5\varphi_{m}}$	(13)	For *φ* < *φ_m_*,	[124]
Moderately concentrated NE + NS	$\mu_{r}=\frac{10\left(\lambda +1\right)+3\varphi \left(5\lambda +2\right)}{10\left(\lambda +1\right)-2\varphi \left(5\lambda +2\right)}$	(14)	*λ*-viscosity ratio, *φ* < 0.02 Usually poor prediction	[125]

**Table 2:** Reported behavior of non-newtonian nanoemulsions (* commercial paraffins).

Dispersed/Continous Fluid/Surfactant/Nucleating Agent	Rheological Behavior	Other Parameters (Shear Rate, ND Concentration)	References
n-alkanes/water	Shear-thinning	80–1000 s^−1^	[142]
Water/n-decane/sorbitan monolurate	Shear-thinning	100–1000 s^−1^; >20%	[102]
Rubitherm^*^ RT10/water	Pseudo-plastic	200 s^−1^; 15–75%	[11,143]
Paraffin/water/PEG-PVA/graphite	Slight shear-thinnning	20%	[144]
* RT70HC/water/Na dodecyl sulphate	Strong non-newtonian	10%	[145]
Paraffin/water	Pseudo-plastic	>40%	[131]
n-hexadecane/water n-octadecane/water	Slight shear thinning	<1000 s^−1^; >40%	[58]
* RT21HC (10 wt.%)/water * RT21HC (2%; 4%)/water * RT21HC + RT55/water	Slight shear thinning; newtonian; newtonian	30–60 s^−1^, 10%; 2%, 4%; 3.6% + 0.4%	[30]

**Table 3:** Variation in physico-thermal properties with different variables for nanoemulsions.

Property	Droplet Concentration	Temperature	Effect on Heat Storage/Rate	References
Density	Increase	Decrease	Positive	[99,100]
Viscosity	Increase	Decrease	Negative	[30,58,102]
Thermal conductivity	Increase	Increase/Decrease	Positive	[57,102]
Specific heat/latent heat	Increase	-	Positive	[57,58]
Surface tension	Decrease	-	Positive	[30]

**Table 4:** Experimentally verified heat transfer correlations in forced-convection conditions (*Nu* and heat transfer, *h*, calculation).

Nanoemulsion	Geometry/Flow Regime	Heat Transfer Correlation		References
Several solid–liquid suspensions	Circular pipe/ laminar, turbulent flow	$Nu=0.202Re^{0.675}Pr^{0.4}{\left(d/d_{p}\right)}^{0.092}{\left(\mu_{susp}/\mu \right)}^{-1.95}$ Re=27,000−120,000; Pr=2.1–3.4; $\frac{\mu_{susp}}{\mu }=1.17-1.83$;$\;\frac{d}{d_{p}}=182-512$; *φ* = 0.005–0.03; accuracy 20%	(53)	[193]
MPCM	Circular pipe/ laminar, turbulent flow	$Nu=0.016Re^{0.88}Pr^{1/3}{\left(\mu_{susp}/\mu \right)}^{0.14}$ Re=8000–50,000; $0.01\leq \dot{m}\leq 0.1$; $0.0024\leq d_{p}/d\leq 0.071$; accuracy ±15%	(54)	[194]
Beewax/water/	Circular tube (ST)	$Nu=1.86{\left(\frac{\mu_{fl}}{\mu_{w}}\right)}^{0.14}{\left(Re\;Pr\frac{d}{L}\right)}^{1/3}$	(55)	[192]
Beewax/water/Paraffin/water/SDS	Circular tube laminar, turbulent (DB); annular tube/turbulent flow	$Nu=0.023Re^{0.8}Pr^{0.4}$	(56)	[140,192]
		$Nu_{ann}=0.86Nu_{DB}\frac{d_{e}^{0.16}}{d_{i}}$	(57)	
Tetradecane/water	Double coiled tube heat exchanger/laminar flow	$Fo=aRe^{*b}\;\;$; *a,b* coefficients dependent on temperature; $Re^{*}=8^{1-n}{\left(\frac{3n+1}{4n}\right)}^{-n}\left(\frac{\rho v^{2-n}d^{n}}{\mu }\right)$ *n* is the power law index from the viscosity rheological equation	(58)	[191]
Water/PAO	Minichannel/transition, turbulent flow (G)	$Nu=\frac{\left(f/8\right)\left(Re-1000\right)Pr}{1+12.7{\left(\frac{f}{8}\right)}^{1/2}\left(Pr^{2/3}-1\right)}$ $f={\left(1.82lnRe-1.64\right)}^{-2}$ $3000<Re<5\times 10^{4}$	(59)	[195]
Paraffin/water	Laminar flow	$\frac{h}{h_{o}}=0.023Re^{0.8}{\left(\varphi \frac{∆H}{c_{p}T}+1\right)}^{1/3}$	(60)	[196,197]
MPCM phase change	Laminar flow	$Nu=0.8148\times 10^{-4}Re^{0.4593}Pr^{0.4836}St^{-0.1277}{\left[\left(L_{1}+L_{2}\right)/D\right]}^{0.3059}$ $\left(L_{1}+L_{2}\right)$ is the length of the phase change region; 60≤Re≤2200; 12≤Pr≤73; 0.05≤φ≤0.276	(61)	[194]
MPCM phase change	Turbulent flow	$Nu=4.8527\times 10^{-4}Re^{0.7733}Pr^{2.7941}St^{0.3159}{\left[\left(L_{1}+L_{2}\right)/d\right]}^{-0.333}{\left(\mu_{d}/\mu \right)}^{-2.4349}$ 2100≤Re≤3500; 13≤Pr≤15; 0.05≤φ≤0.1	(62)	[194]

**Table 5:** Parameters that influence heat transfer in nanoemulsion slurries.

Parameter of Influence	Influence on Heat Transfer: Positive/Negative	Mechanism of Enhancement	References
Effective thermal conductivity	+	Possibly increased local convection	[198]
Effective heat capacity	+	Increased bulk heat energy storage	[58]
Nanodroplet concentration	+	Increased bulk thermal properties and turbulence	[57,58]
Reynolds number	+	Enhanced turbulence	[139,192]
Stefan number	+/−	Combined effect of parameters	[189]
Prandtl number	+	Combined effect of thermal properties	[192,195]
Grashof number	+	Enhanced natural convection	[207,208,209]
Phase change temperature range	−	Better use of phase change energy	[189]

## Data Availability

Not applicable.

## Nomenclature

*E*	thermal energy, W
*m*	mass, kg
*c*_*p*_	specific heat, J/kg K
*T*	temperature, K
*H*	entalpy, J/kg
ρ	density, kg/m^3^
φ	phase volume fraction, m^3^/m^3^
μ	dynamic viscosity, Pa·s
*λ*	viscosity ratio
*K*	aggregation coeficient
*C*	coefficient
*δ*	nanolayer thickness, m
*a*	particle radius, m
*k*	thermal conductivity, W/mK
$\dot{q}$	heat flow per unit length, W/m
*t*	time, s
*V*	voltage, V
ω	frequency, Hz
*R*	wire radius, m
*P*	applied electric power, W
*L*	length, m
γ	ratio between the interfacial thermal resistance and particle size
D	diffusion coefficient, m^2^/s
Φ	phase mass fraction, kg/kg
*ct*	calibration constant
$\hat{T}$	modulation period, s
*c*	calorimetric constant
*A*	area, m^2^
σ	surface tension, N/m
*r*	radius of curvature of the droplet surface, m
*p*	pressure, N/m^2^
*Mo*	Mouromtseff number
FOM	figure of merit
*η*	cooling capacity
α	thermal diffusivity, m^2^/s
ε	thermal effusivity, Ws^1/2^/m^2^K
*St*	Stefan number
*Gr*	Grashof number
*l*	characteristic length, m
g	gravitational acceleration, m/s^2^
β	volumetric thermal expansion coefficient, K^−1^
*Re*	Reynolds number
*v*	velocity, m/s
*Pr*	Prandtl number
*Nu*	Nusselt number
*h*	heat transfer coeeficient, W/m^2^K
*Fo*	Fourier number
d	pipe diameter, m
*n*	power law index from the viscosity rheological equation
f	Fanning factor
Δ	variation
Subscripts:	
*SHS*	sensible heat storage
*i*	initial
*f*	final
*LHS*	latent heat storage
*mt*	melting
*NE*	nanoemulsion
*c*	continuous
*r*	relative
*susp*	suspension
*1,2,3*	first, second, third order
*solv*	solvated
*eff*	effective
*sol*	solvation
*m*	maximum packing
*n*	nanofluid
*p*	particle
*sph*	spherical
*cyl*	cylindrical
*M*	Maxwell
*d*	dispersed
*B*	Brownian
*PCM*	phase change material
*b*	blank
*amp*	amplitude
*fr*	freezing
*W*	water
*t*	tube material
*s*	solid
*l*	liquid
*pr*	probe
*fl*	bulk fluid
*w*	wall
*ann*	annular
*DB*	Ditus–Boelter
Constants:	
*K_B_*	Boltzmann constant, J/K
